# Diversity and interactions of microbial functional genes under differing environmental conditions: insights from a membrane bioreactor and an oxidation ditch

**DOI:** 10.1038/srep18509

**Published:** 2016-01-08

**Authors:** Yu Xia, Man Hu, Xianghua Wen, Xiaohui Wang, Yunfeng Yang, Jizhong Zhou

**Affiliations:** 1 Environmental Simulation and Pollution Control State Key Joint Laboratory, School of Environment, Tsinghua University, 100084, Beijing, P.R. China; 2 Institute for Environmental Genomics and Department of Botany and Microbiology, University of Oklahoma, Norman, OK, USA

## Abstract

The effect of environmental conditions on the diversity and interactions of microbial communities has caused tremendous interest in microbial ecology. Here, we found that with identical influents but differing operational parameters (mainly mixed liquor suspended solid (MLSS) concentrations, solid retention time (SRT) and dissolved oxygen (DO) concentrations), two full-scale municipal wastewater treatment systems applying oxidation ditch (OD) and membrane bioreactor (MBR) processes harbored a majority of shared genes (87.2%) but had different overall functional gene structures as revealed by two datasets of 12-day time-series generated by a functional gene array-GeoChip 4.2. Association networks of core carbon, nitrogen and phosphorus cycling genes in each system based on random matrix theory (RMT) showed different topological properties and the MBR nodes showed an indication of higher connectivity. MLSS and DO were shown to be effective in shaping functional gene structures of the systems by statistical analyses. Higher MLSS concentrations resulting in decreased resource availability of the MBR system were thought to promote positive interactions of important functional genes. Together, these findings show the differences of functional potentials of some bioprocesses caused by differing environmental conditions and suggest that higher stress of resource limitation increased positive gene interactions in the MBR system.

Wastewater treatment plants (WWTPs) are the largest applications of bioprocess engineering used for domestic and industrial wastewater treatment, with microbial consortia playing the central role. System-level functions (e.g., biodegradation and mineralization of organic pollutants, and nitrogen and phosphorus cycling) and ecosystem stability are accomplished through the growth, activities and interactions of vastly diverse microbial populations. As a unique artificial microbial ecosystem which is chemically and physically well-defined, WWTPs are considered as a fertile testing ground for a range of fundamental ecological questions^1^.

Environmental heterogeneity, defined as spatial and temporal variation in the physical, chemical and biological environment, is a fundamental property of ecosystems^2^. Its role in shaping microbial community diversity and composition is commonly valued and an intriguing topic. WWTPs are meant to provide proper environmental conditions to maintain the abundances of microbial populations, especially those functionally important groups, within normal levels for system performance and stability. Therefore, numerous studies focus on the relationship between microbial community diversity and environmental conditions mainly based on 16S rRNA genes or specific functional genes. They reveal the differences in microbial diversity and composition resulting from the differentiations of, such as, influent chemical oxygen demand (COD inf) concentrations^3^, dissolved oxygen (DO) concentrations^4^ and solid retention time (SRT)^5^. Specifically, it has been suggested that the fixed volume bioreactors operated at high SRTs will be highly saturated with organisms that are capable of efficiently utilizing scarce resources (‘*K*-strategists’), while low SRTs will help enrich for fast-growing organisms that are adapted for high resource utilization (‘*r*-strategists’)^6^. Some *K*-strategists such as *Nitrospira* sp., *Nitrosomonas* sp., and taxa phylogenetically associated with *Planctomycetes* and *Chloroflexi*, were shown to be present only at SRTs ≥ 12 days in a wastewater treatment system where different SRTs (30 d, 12 d and 3 d) and correspondingly different mixed liquor suspended solid (MLSS) concentrations were applied at different stages^5^.

The description of inventory diversity is not sufficient for a complete picture of the microbial ecology of an ecosystem. Microbial interactions are also a key topic of microbial ecology, through which diverse communities can perform better than the best performing species alone. With high-throughput techniques enjoying an overwhelming popularity and a large amount of data accumulated, microbial interactions of taxonomic groups inhabiting WWTPs were revealed very recently through their spatial^7^ and temporal^8^ co-occurrence patterns. Clarifying the effect of environmental variations on microbial interactions is necessary for a systematic understanding of microbial ecology of WWTPs, which may also enable engineers to structure such communities more efficiently.

Currently, municipal WWTPs are mainly designed to achieve the removal of organic carbon, nitrogen and phosphorus. Membrane bioreactors (MBRs) and conventional activated sludge (CAS) bioreactors (including the systems applying modified activated sludge processes, such as sequential batch reactor (SBR), anaerobic-axoic-oxic (A^2^O), and oxidation ditch (OD)) are commonly applied treatment processes with differing operational parameters. MBRs are typically operated at higher MLSS concentrations, longer SRTs with membrane filtration, higher DO concentrations resulting from higher aeration intensity for creating higher shear forces to control membrane fouling. Higher MLSS concentrations resulting in lower food to microorganism (F/M) ratios expose MBR communities to higher nutrient stress because of resource limitation. Microbial diversity and interactions of MBRs may be thus different from those of CAS bioreactors. Moreover, although full-scale MBR and CAS systems for municipal wastewater treatment are both meant to support the slow-growing populations like nitrifiers, certain *K*-strategists may indeed be advantaged in the MBRs with longer SRTs. Different DO concentrations may also cause specific differences in the composition of microbial communities. Research on the differences of microbial populations of CAS and MBR systems should be a good way to understand the correlations between environmental gradients and microbial ecology. Also, characterizing the microbial communities present in engineered systems is valuable to understand the function of the system.

Existing research shed light on the differentiations of taxonomical structures^9^,^10^ and diversity of certain functional genes (e.g. nitrification and denitrification genes) of CAS and MBR systems^11^. However, to directly address microbial functional potentials related to system processes, it is crucial to examine more categories of microbial functional signatures such as structural genes relevant to metabolic pathways, energetics and regulatory circuits^12^. Apart from microbial diversity, the ecological interactions of microbes in MBR systems have never been reported, let alone the interpretation of how possible microbial interactions of ecosystems differ with gradients of substrate availability.

In this study, we focused on two full-scale MBR and OD systems operated parallelly in one wastewater treatment plant with identical influent. According to the data issued by the Ministry of housing and urban-rural development of China, by the end of 2013, the OD technology had been the mostly widely used treatment process in municipal wastewater treatment plants (MWTPs) of China (accounting for 26.7% in number). The cumulative treatment capacity of OD systems had ranked the second in China (accounting for 25.2%), following that of the A^2^O systems (accounting for 36.5%). Investigations of the two treatment systems are important for understanding not only the correlations of environmental gradients and microbial ecology, but also full-scale MWTP functioning. For each system, 12 concecutive daily samples were collected, and overall functional gene diversity directly linked to microbial functional potential of each sample was analyzed by using a comprehensive functional gene microarray – GeoChip 4.2. Possible functional gene interactions were revealed through association network construction by using a random matrix theory (RMT) algorithm^13^. The specific scientific questions we address are: (1) what the functional gene diversity and composition of the MBR and OD systems are; (2) how important functional genes in each system are possilby linked; (3) how environmental variables affect the temporal assembly patterns of overall functional genes. We believe that this presents a first investigation unveiling the differentiations of functional gene diverisity and possible microbial interacions of MBR and OD systems. Also it will give a useful insight of the effect of environmental conditions on the ecology of the microbial world.

## Results

### Bioreactor performance and operational conditions

The two systems treated identical municipal wastewater. The differences of treatment processes and environmental conditions of the two systems are shown in Supplementary Table S1 online. COD inf concentrations were 399.4 ± 77.7 mg/L; influent total nitrogen (TN inf) concentrations were 44.2 ± 5.9 mg/L; influent total ammonia (NH_4_^ + ^-N inf) concentrations were 34.1 ± 5.4 mg/L and influent total phosphorus (TP inf) concentrations were 5.1 ± 1.3 mg/L (Supplementary Table S2 online). Bioreactor pH, temperature and DO concentrations of the MBR system were 6.92 ± 0.07, 19.1 ± 0.7 °C and 2.95 ± 0.46 mg/L, respectively, and those of the OD systems were 7.05 ± 0.10, 19.3 ± 0.7 °C and 1.69 ± 0.51 mg/L, respectively (Supplementary Table S3 online). MLSS concentration of the OD system was mantained around 4,500 mg/L, and that of the MBR system was around 7,000 mg/L. The SRT of the MBR system was about 20.5 d and that of the OD system was approximately 16.2 d. The two systems showed relatively stable treatment efficiencies. The MBR system showed a higher COD removal efficiency, while the OD system had a better TN removal performance (Supplementary Table S3 online).

### Overall functional gene diversity of the two systems

A total of 36,420 functional genes were detected, with 35,060 genes present in the OD system and 33,117 genes present in the MBR system. The genes were involved in 16 bioprocesses, such as carbon-cycling, nitrogen-cycling, phosphorus-cycling and bacterial phage. The α-diversity indices (Table 1) suggested that the two systems had high functional gene diversity, and the OD system exhibited higher microbial diversity. A majority of functional genes (87.2%) were shared by the two systems, involved in each category. In spite of the shared genes, each system possessed some unique genes associated in each category. The genes only present in the MBR system accounted for 3.7% and the value was 9.1% for the OD system.

**Figure 1:**
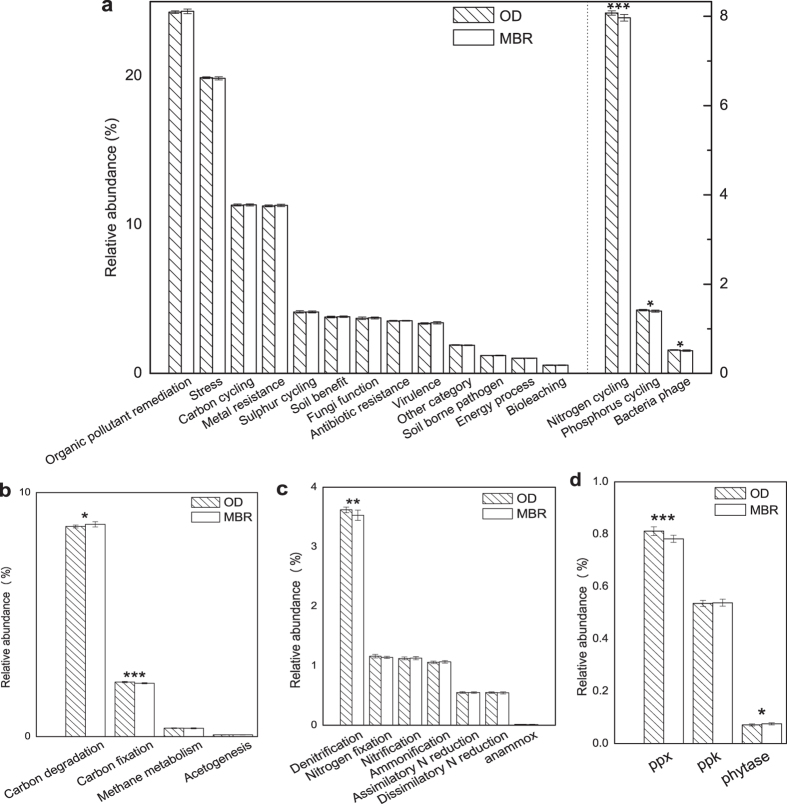
Relative abundances of the functional genes involved in certain bioprocesses. (**a**) each category of functional genes, (**b**) each subcategory of carbon cycling genes, (**c**) each subcategory of nitrogen cycling genes, and (**d**) each subcategory of phosphorus utilization genes. Error bars represent the standard deviation of the relative abundances of each category or subcategory of genes in the 12 samples from the same system. Significant differences between the systems indicated by repeated measures analysis of variance (ANOVA) are indicated by asterisk above the bars, ‘*’ (*P* < 0.05), ‘**’ (*P* < 0.01), ‘***’ (*P* < 0.001).

The two systems displayed similar relative abundance distributions of each category of functional genes. The OD system showed slightly higher overall relative abundances of the genes associated with bacterial phage (*p* < 0.05, absolute value of Cohen’s *d* = 0.89), nitrogen cycling (*p* < 0.001, absolute value of Cohen’s *d* = 1.31) and phosphorus utilization (*p* < 0.05, absolute value of Cohen’s *d* = 0.99) (Fig. 1a).

### Diversity of the genes related to key bioprocesses of wastewater treatment

To clarify the details of the functional potential differentiations of the two systems, we focused on the diversity of three important functional gene categories - carbon cycling, nitrogen cycling and phosphorus utilization, as the removal of carbon, nitrogen and phosphorus pollutants are the main task of MWTPs.

A total of 4,129 genes associated with carbon cycling were detected, with 3,976 genes present in the OD system and 3,735 genes present in the MBR system. Among them, carbon degradation genes were predominant, accounting for 76.6%. These genes played roles in the removal of several polysaccharides such as starch, cellulose and chitin. Carbon degradation genes were slightly more abundant within the MBR system than in the OD system (*p* < 0.05, Cohen’s *d* = 0.95) (Fig. 1b).

A total of 2,983 genes associated with nitrogen cycling were detected, consisting of the genes involved in ammonification (12.9%), nitrification (14.2%), denitrification (43.4%), anammox (0.2%) and others (29.3%). The detected 424 nitrification genes consisted of 408 *amoA* genes and 16 *hao* genes, of which 55.9% were from putative heterotrophic nitrifiers such as *Pseudomonas putida*. In addition, 11.3% of the nitrification genes were from the order of *Nitrosomonadales*, including *Nitrosomonas* (seven genes), *Nitrosospira* (15 genes) and *Nitrosovibrio* (one gene), which are the commonly known ammonia oxidizing bacteria (AOB) in WWTPs. The genes from *Nitrosospira* were slightly of higher relative abundance within the MBR (*p* < 0.05, absolute value of Cohen’s *d* = 0.88), with the percentage change of relative abundances being 8.3%. Also, archaeal genes were detected, accounting for 18.4% of the nitrification genes. In the 1295 genes detected associated with denitrification, there were 88.5% from uncultured isolates. The relative abundance of denitrification genes of the OD system was slightly higher than that of the MBR system (*p* < 0.01, absolute value of Cohen’s *d* = 1.09) (Fig. 1c).

A total of 528 genes associated with phosphorus utilization were detected, including *ppk* genes (37.3%), *ppx* genes (57.6%) and *phytase* genes (5.1%). Polyphosphate kinase (ppk) is responsible for polyphosphate (polyP) synthesis and *ppk* genes did not show significant differences in relative abundances within the two systems (Fig. 1d). Exopolyphosphatase (ppx) is a highly processive enzyme catalyzing the anaerobic hydrolysis process of terminal residues of long-chain polyP to phosphate (Pi). The relative abundances of *ppx* genes were significantly higher within the OD system than in the MBR system (*p* < 0.001, absolute value of Cohen’s *d* = 1.39) (Fig. 1d). Phytase catalyzes the step-wise release of phosphate from phytate, the principle storage form of phosphorus in plant seeds and pollen^14^. *Phytase* genes were of slightly higher relative abundance within the MBR system than in the OD system (*p* < 0.05, Cohen’s *d* = 0.96) (Fig. 1d).

### Functional gene structures of the two systems

**Figure 2:**
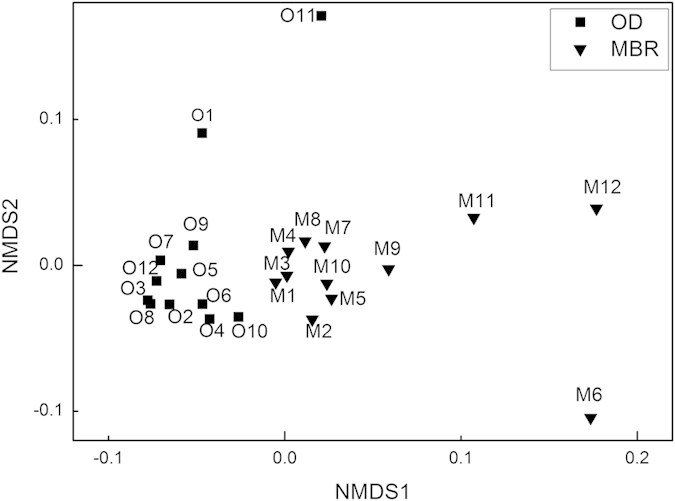
Nonmetric multidimensional scaling (NMDS) analysis of the overall functional genes of the microbial communities in the investigated 24 samples. The samples from the same wastewater treatment system grouped.

The nonmetric multidimensional scaling (NMDS) result revealed that the samples from each system grouped (Fig. 2). Moreover, dissimilarity tests indicated that the two systems showed distinct structures of overall functional genes, carbon degradation genes, nitrogen cycling genes and phosphorus cycling genes (Table 2). No significant correlations between pollutant (COD, TN and NH_4_^ + ^-N) removal efficiencies and overall functional genes structures were suggested by the mantel test. As the phosphorus removal of the systems did not rely merely on biological processes, phosphorus removal efficiencies were not directly linked to functional gene structures when doing the analysis.

In each system, the samples did not show obvious patterns in the NMDS plot and the abundances of each category of functional genes had small variations during the 12 days (Fig. 1), indicating that there was no obvious daily succession of overall functional genes.

### Effective environmental variables shaping functional gene structures

**Figure 3:**
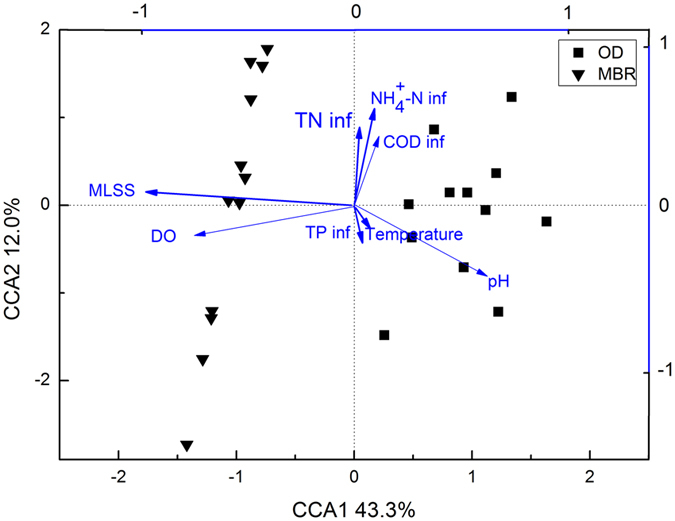
Canonical correspondence analysis (CCA) of the signal intensities of overall functional genes and environmental attributes. The amount of explained cumulative variation for axis 1 and axis 2 was 43.3% and 12.0%, respectively.

A canonical correspondence analysis (CCA) was performed to evaluate the effects of nine environmental variables on overall functional gene structures, including SRT, MLSS concentrations, DO concentrations, pH and temperature of the bioreactors, COD inf concentrations, NH_4_^ + ^-N inf concentrations, TN inf concentrations and TP inf concentrations. SRT was evaluated as a redundant factor by its variance inflation factor (VIF) value and thus eliminated when with the presence of MLSS concentration. A CCA ordination biplot of the overall functional genes and the remaining environmental variables arranged along the first two ordination axes was generated by constraining the axes to be linear combinations of environmental variable scores (Fig. 3). MLSS and DO were evaluated to be effective factors shaping the overall functional gene structures of the two systems in the CCA plot. The mantel test result indicated significant links between the overall functional gene structures and MLSS (*r* = 0.4281, *P* = 0.001), SRT (*r* = 0.4281, *P* = 0.001) and DO (*r* = 0.2795, *P* = 0.001). No significant correlations between functional gene structures, and wastewater characteristics and bioreactor temperature and pH were revealed by the mantel test (data not given).

### Possible interactions of core genes involved in the cycling of carbon, nitrogen and phosphorus

The coexistence of a number of genes was noticed within each system. In the OD system, there were 50.9% genes present in all of the 12 daily samples. For the MBR system the value was 47.6%. Detailed information of the coexistent genes in the 12 sampling days of each system is shown in Supplementary Table S4 online. We consider these coexistent genes to be the core functional genes of each system. Possible interactions of these core genes implicated in carbon, nitrogen and phosphorus cycling were revealed by network construction based on a RMT algorithm. Consequently, a network of 1,116 nodes and 1741 links (4.2% negative and 95.8% positive) was obtained for the MBR system and a network of 1,426 nodes and 1,462 links (9.4% negative and 90.6% positive) for the OD system.

**Figure 4:**
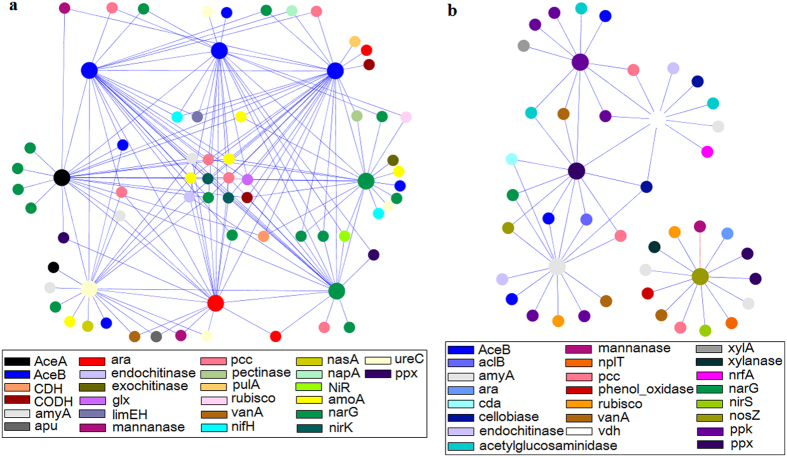
Different network interactions of key functional genes in the two systems. (**a**) Network interactions of the functional genes the connectivities of which ranked top five in the MBR system. (**b**) Network interactions of the functional genes the connectivities of which ranked top five in the OD system. Colors of the nodes indicate different functional genes. A blue line indicates a positive interaction between two nodes, and a red line indicates a negative one. The nodes in this figure are carbon cycling-, nitrogen cycling- and phosphorus utilization- related genes.

The two networks exhibited general topological features of ecological networks including scale free, small world and modularity (Supplementary Text S1 online). The topological structures of the two networks differed significantly, including average geodesic distance, average clustering coefficient, modularity and transitivity (Table 3). Average connectivity, average clustering coefficient and transitivity of the MBR network were higher than those of the OD network. The links between the nodes whose connectivity ranked top five in each network were shown in Fig. 4. Only 22.0% (458) of the nodes were shared by the two networks. Significant differences were observed in connectivity (*p* = 9.51 × 10^−6^) and clustering coefficients (*p* = 2.19 × 10^−8^) of these shared nodes in the two networks, as revealed by paired *t* tests.

There were 200 modules detected within the MBR network and 295 modules detected in the OD network. In each module, various categories or subcategories of functional genes were included. For each network, most of the nodes were evaluated as peripheral nodes, which only have a few links almost always to the nodes within their modules and represent specialists from an ecological perspective. Few of the nodes were defined as module hubs in each system, which were highly connected to several nodes in their own modules. In the MBR network 12 module hubs were detected, consisting of the genes involved in carbon degradation (66.7%), nitrification (8.3%), denitrification (8.3%), nitrogen fixation (8.3%) and dissimilatory N reduction (8.3%). In the OD network, 16 module nodes were detected, consisting of the genes related to carbon degradation (37.5%), carbon fixation (12.5%), phosphorus utilization (18.8%), denitrification (12.5%), nitrogen fixation (12.5%) and dissimilarity N reduction (6.3%). Only one connector (highly linked to several modules) associated with nitrogen ammonification was detected within the MBR network, and no connectors were detected within the OD network. From an ecological perspective, module hubs and connectors are generalists. None of the module hubs or connectors of the two networks were the same. The details of the module hubs and connectors of each network are shown in Supplementary Table S5 online.

For each network, the relationships between node connectivity and seven environmental variables (DO, pH and temperature of the bioreactors, COD inf, NH_4_^ + ^-N inf, TN inf and TP inf) were examined. In the OD network, none of the gene significance (GS) of the environmental variables showed a significant correlation to node connectivity. In the MBR network, the GS of bioreactor pH showed a weak linkage to node connectivity (Supplementary Table S6 online).

## Discussion

A high similarity of overall functional genes of the two systems was detected. It is known that the removal of wastewater-borne organic and inorganic pollutants (for example, PO_4_^3−^ and heavy metals) relies heavily on the coexistence of several key microbial groups and the occurrence of particular microbial species^7^,^15^. The two systems were both designed for the removal of organic carbon, nitrogen and phosphorus pollutants. Several overlapped functional genes may participate in each bioprocess in both systems. Besides, inoculum sludge from the OD system was used for the start-up of the MBR system. Original MBR populations came from the OD community. Also, identical wastewater would facilitate the community similarity, as the bioreactor communities were exempt from the differences in substrate composition and source community, compared to the populations of the systems receiving differing influents. Moreover, identical wastewater and location leaded to similar temperature and pH of the bioreactors, providing the community with similar physical-chemical conditions. Similar results were obtained in the conventional enhanced biological phosphorus removal (EBPR) and MBR plants in Denmark, which showed a majority of shared 16S rRNA genes^10^.

However, slight differences in the abundances of certain categories and subcategories of functional genes of the two systems were shown. They may result from the common differences in environmental conditions of these two kinds of treatment processes. MBRs were suggested to have a better disinfection capability than CAS systems^16^. Phage removal in a MBR was suggested to be largely achieved by biofilm accumulation on the membrane surface, which physically reduced the membrane pore size, chemically adsorbed phages, and biologically allowed the predation of phages by other microorganisms^17^. Thus, bacterial phages in the MLSS of the membrane tanks (the MBR samples) may be of a low abundance, and they may be less abundant than those of the OD samples. The longer SRT of the MBR system might allow the enrichment of some microbes responsible for the degradation of high-molecular-weight compounds such as polysaccharide and protein^18^. Besides, the higher concentration of MLSS in the MBR system may lead to an increase of the concentration of colloidal material, which presents a new feed source for the microorganisms able to scavenge these compounds. This is verified by the fact that the concentrations of polysaccharides in the supernatant of the membrane tanks of the MBR system (2.4-6.5 mg/l) were higher than those of the aeration tanks of the OD system (1.3-5 mg/l) during April, 2011 to March, 2012 (unpublished data). Thus, the carbon degradation genes may be more abundant within the MBR samples. Possible reasons for the differences in the abundances of nitrogen cycling genes are as follows. The higher abundance of the nitrification genes from *Nitrosospira* in the MBR system might result from the lower F/M ratio, as *Nitrosospira* were suggested to be favored under relatively low concentrations of substrates, compared to *Nitrosomonas*^19^. The higher abundance of denitrification genes within the OD system might be due to the lower DO concentration and the higher F/M ratio. The former may promote denitrification and the latter may allow the denitrifiers to access more readily biodegradable carbon sources. Possible explanations of the differences in the abundances of phosphorus utilization genes are listed below. The higher abundance of *ppx* genes within the OD system may mainly result from the lower DO concentration, which may benefit the anaerobic hydrolysis process of terminal residues of long-chain polyP to phosphate (Pi). It was suggested that phytase is not required for balanced growth of bacterial cells, but may be synthesized in response to a nutrient or energy limitation^20^. The higher abundance of *phytase* genes of the MBR system may be due to a severer situation of the nutrient or energy limitation the MBR community was faced up with. These significant differentiations in the abundances of certain functional gene groups (for example, higher abundance of carbon degradation genes in the MBR system, and higher abundance of denitrification genes in the OD system) revealed specific effects of environmental gradients on the functional potential of specific bioprocesses. They may explain the observed differences of system performance in COD and TN removal to some extent. Differing structures of overall functional genes and specific categories of functional genes of the two systems were also revealed. This may result from the existence of unique genes of each system and abundance differentiations of certain genes. No significant linkages between system functioning and overall functional genes structures were detected, however. The possible reasons might be that the unique functional genes of each system or the genes with differing abundances may not be so tightly linked to removals of COD, TN and NH_4_^+ ^-N.

In addition to the differentiations of functional gene abundances and structures, tighter interactions/coupling, especially tighter positive (facilitative) interactions within the MBR community was suggested by the higher average connectivity, average clustering coefficient, transitivity and positive interaction percentages of the MBR network. This may result from the lower F/M ratios of the substrates of carbon, nitrogen and phosphorus in the MBR system. For the MBR populations, amelioration of the stress of substrate scarcity by collaborations with the neighbors may be a good choice. For example, the carbon degradation groups could increase the availability of readily degradable organic carbon for denitrifiers. Thus, the organic carbon degradation population and denitrifiers may be more tightly connected in the MBR than in the OD system. Similarly, facilitation could be expected to be the dominant net outcome at moderate levels of stress driven by resource in plants^21^ and it was noticed that positive interactions between plant species were dominant at moderate level of rainfall stress^22^. No overlaps of the generalist nodes within the two networks were detected, indicating that important roles within or between modules of the MBR and OD systems were played by different nodes (genes).

The CCA unveiled that MLSS and DO were main factors shaping the functional gene structures of the two systems, which was verified by the mantel test. As is discussed above, the higher MLSS of the MBR system, which resulted in lower F/M ratios, may favor *K*-strategists like *Nitrosospira*, select against denitrifers and promote *phytase* genes in the MBR system. Also, it resulted in more polysaccharides of the supernatant, which may contribute to the higher abundance of carbon degradation genes of the MBR system. The higher aeration rates of the MBR system may select against denitrifiers and *ppx* genes. Due to the numerical correlation between SRT and MLSS concentrations in doing the CCA, SRT was not evaluated as a redundant variable when with the presence of MLSS and excluded from the CCA plot. On the other hand, the significant correlation between SRT and the functional gene structures in the mantel test indicates that SRT may exert some effects on the functional gene structures. Specifically, as is discussed above, the longer SRT of the MBR system might help promote some organisms able to degrade polysaccharides, for example *Chloroflexi*^5^,^23^. It may be an explanation of the higher abundance of carbon degradation genes. Short SRTs were shown to have impacts on microbial communities of wastewater treatment plants in previous research. A system operated at a SRT of 3d was indicated to harbor no *Nitrospira* sp. and *Nitrosomonas* sp.^5^. Another system operated with SRTs of 10 d, 3 d and 5 d was shown to have more diverse microbial populations when with a higher SRT^24^. However, differing SRTs of the two systems did not lead to significant differences of nitrification gene abundances (Fig. 1c). The possible reason might be that the SRTs of the two systems (20.5 d and 16.2 d) could both allow the nitrifiers to thrive. Besides the effects of operational parameters on the functional gene structures, a low percentage of shared nodes, differing topological structures and different topological roles of the nodes in the two networks indicated that operational parameter differentiations also altered possible microbial interactions of the two systems. The higher MLSS of the MBR system increased the resource limitation stress and may, thus, increase positive interactions between the functional genes associated with carbon, nitrogen and phosphorus cycling. The possible impacts of treatment processes and environmental variables on the diversity and interactions of the functional genes in the two systems are summarized in Supplementary table S1 online.

Wastewater characteristics, and bioreactor temperature and pH, which were vulnerable to influent variations, played relatively small roles in shaping the overall functional gene structures. In addition, no significant or only few weak links between these variables and the topology of each network revealed their inability in affecting the possible microbial interactions. The reason might be that the two systems were with small wastewater characteristics fluctuations and basically stable temperature and pH during the sampling days. Besides, some unmeasured influential variables could also possibly contribute to significant changes in functional gene diversity and affect possible interactions of core functional genes in each system.

When long-term sampling is implemented, larger gradients in other parameters, for example, bioreactor temperature and pH, are covered. The main environmental variables that affect microbial ecology might be different. A long-term investigation on a single WWTP using high-throughput sequencing indicated the importance of temperature and salinity in driving the seasonal dynamics of the genera with significantly changed abundances during an over 4-year period^25^. Another study indicated a lack of strong correlations between environmental factors and several persistent operational taxonomy units (OTUs) in a WWTP. Environmental conditions (mainly SRT and inorganic nitrogen) partially explained the phylogenetic variances and indirectly influenced bacterial assembly^8^. These studies suggest that the effective variables shaping microbial community structures may be case-specific. However, few long-term investigations on the communities of MBRs and activated sludge systems have been reported. Such research did not discuss which variables were predominant in leading to the observed differentiations of microbial diversity either^11^. Long time-series sampling (for example, monthly, seasonal and yearly) of the communities in MBRs and activated sludge systems are suggested to be conducted by further studies to enable profound discussions on the correlation between microbial ecology and environmental gradients, with big environmental gradients in both physical-chemical and operational parameters covered.

In sum, in this study we showed that the differences in operational parameters (MLSS, SRT and DO) of two parallelly operated MBR and OD systems indeed affected not only the functional potentials of certain bioprocesses but the microbial interactions. These findings give a useful insight of the effect of differing environmental conditions on microbial ecology. WWTPs are well controlled microbial ecological systems, but we only know a tip of the iceberg of them. Further studies on these systems targeting at answering key ecological questions and understanding the systems for a better design and operation of them are of great significant.

## Methods

### Location Description and Sampling

The two full-scale wastewater treatment facilities are located in a WWTP in Wuxi, Jiangsu Province of China. One of them is an orbal OD, and the other is a MBR coupled with anaerobic-anoxic-oxic process (A^2^O-MBR). They treat identical wastewater (domestic : industrial = 0.6 : 0.4) at the same scale (50,000 m^3^/d) and the MBR sludge was originally inoculated with the OD sludge. The removal of organic carbon and nitrogen pollutants of the systems were achieved through biological processes, and the removal of phosphorus of them were through both biological processes and chemical precipitation. They had been operated with good treatment efficiency and stability at least for one year and three months before sampling^26^,^27^.

MLSS samples were taken from the the aerobic zones of the OD system and from the membrane tanks of the A^2^O-MBR system once a day for 12 concecutive days during April 10 to 21, 2011. On each day, 50 ml MLSS was collected at each site. Each sample was dispensed into a 50 mL sterile Eppendorf tube and centrifuged at 14,000 *g* for 10 min. The pellets were stored at −80° C for analysis. Daily measurement was done to decide the pollutant concentrations of 24-h composite samples of influents and effluents, and temperatures, pH, and DO concentrations of the aeration zones of each system. The approximate values of the MLSS concentrations and SRT were provided by the plant staff.

### DNA Extraction and Purification

Microbial genomic DNA was extracted from the pellets of activated sludge samples through a combination of freezing and sodium dodecyl sulfate (SDS) for cell lysis as previously described^28^. The extracted products were then purified employing the Wizard^®^ SV Genomic DNA Purification Kit (Promega, Madison WI).

### DNA Labeling and Hybridization

1 μg DNA was labeled, purified and dried as previously described^29^. All labeled DNA was resuspended in 10 μL hybridization solution as previously described^30^ and was hybridized with GeoChip 4.2 on a MAUI hybridization station (BioMicro, Salt Lake City, UT, USA) at 42° C with 40% formamide for 16 h. Microarrays were scanned by a ScanArray 5000 Microarray Analysis System (PerkinElmer, Wellesley, MA, USA) at 100% laser power.

### Data Analysis

Signal intensities of the spots were measured with ImaGene 6.0 (Biodiscovery Inc., El Segundo, CA, USA). Data pretreatment was performed online (ieg.ou.edu). The percentages of thermophile probes (negative controls) in each sample were made less than 5% by the removal of low quality spots. The genes detected only in three or fewer samples out of 12 samples from the same system were removed to avoid potential noise. For each sample, intensities of the genes were then transformed to the natural logarithmic form and divided by the mean signal intensity.

Detailed information of the detected genes and their signal intensities is available at the Gene Expression Omnibus (www.ncbi.nlm.nih.gov/geo/, accession number GSE 67307).

To unveil the dissimilarities of microbial communities, three non-parametric multivariate statistical tests including non-parametric multivariate analysis of variance (ADONIS), analysis of similarity (ANOSIM) and multiple response permutation procedure (MRPP) and a NMDS analysis were performed. To test whether the diversity indices and the abundances of each functional gene category and certain subcategories or phylogenetic groups in the two systems differed, repeated measures analysis of variance (ANOVA) was performed. Cohen’s *d* was applied to assess the magnitude of treatment effects and a conventional rule is to consider a Cohen’s *d* of 0.8 as large, suggesting that 79% of the control group would have a score below the subject in the experimental group^31^,^32^. To evaluate the effects of environmental factors on overall functional community structures, a CCA and a mantel test were performed. In CCA, the VIF for each environmental attribute was used to identify multicollinearity between explanatory variables. The factor with a VIF value over 20 was considered to be a redundant constraint and removed. All the above statistical analyses were done with R code (http://www.r-project.org/).

To unveil possible microbial interactions of the genes associated with carbon, nitrogen and phosphorus cycling, two microbial association networks were constructed based on RMT using a comprehensive Molecular Ecological Network Analysis Pipeline (MENAP) (http://ieg2.ou.edu/MENA/)^13^. A similarity matrix of “Pearson correlation in time-series (allow 1 time point lagging)” was applied. For each system, only those genes detected in all the 12-week samples (majority rule) were kept for network construction. This filtering step removed poorly represented functional genes and reduced network complexity^33^. For each network, 100 corresponding random networks were generated, with the same network size and average number of links. The *Z*-test was used to test the differences of the indices between the constructed networks and random networks. To characterize the modularity property, each network was separated into modules by the fast greedy modularity optimization. The topological roles of different nodes were divided into the following four subcategories by within-module connectivity (*z*_*i*_) and among module connectivity (*P*_*i*_): (i) peripheral nodes; (ii) connectors; (iii) module hubs; and (iv) network hubs^34^. For comparison between the network indices of different systems, the Student *t*-test was employed using the standard deviations derived from corresponding random networks. Paired *t*-test was done to make comparisons between the topological structures of the shared nodes of the two networks. The relationships between microbial network topology and environmental characteristics were examined in an indirect way by measuring the correlation between the GS and the connectivity of nodes^12^. Cytoscape_3.2.1 was used for network visualization of the nodes, the connectivity of which ranked top five.

## Additional Information

**How to cite this article**: Xia, Y. *et al*. Diversity and interactions of microbial functional genes under differing environmental conditions: insights from a membrane bioreactor and an oxidation ditch. *Sci. Rep*. **6**, 18509; doi: 10.1038/srep18509 (2016).

## Supplementary Material

Supplementary Information

## Figures and Tables

**Table 1:** Diversity indices of microbial communities of the samples from these two treatment systems.

Sampling day	Richness^a^		H^b^		1/(1-D)^c^		J^d^	
	OD^e^	MBR^e^	OD^e^	MBR^e^	OD^e^	MBR^e^	OD	MBR
1	26250	29360	10.17	10.28	26167.0	29216.3	0.9998	0.9998
2	30820	29339	10.33	10.28	30699.4	29196.8	0.9998	0.9998
3	32338	29872	10.38	10.30	32198.5	29729.3	0.9998	0.9998
4	29202	28354	10.28	10.25	29095.0	28224.0	0.9998	0.9998
5	30275	29196	10.32	10.28	30169.4	29062.6	0.9998	0.9998
6	30802	24547	10.33	10.11	30685.2	24465.1	0.9998	0.9998
7	31133	28590	10.34	10.26	31032.5	28465.6	0.9998	0.9998
8	31068	28824	10.34	10.27	30953.8	28697.8	0.9998	0.9998
9	30172	27582	10.31	10.22	30062.0	27469.6	0.9998	0.9998
10	29663	28458	10.30	10.25	29554.7	28332.3	0.9998	0.9998
11	24743	25487	10.11	10.14	24666.5	25381.2	0.9998	0.9998
12	30740	24046	10.33	10.09	30597.9	23952.5	0.9998	0.9998

^a^Detected gene number.

^b^Shannon-Weiner index, higher number represents higher diversity.

^c^Reciprocal of Simpson’s index, higher number represents higher diversity.

^d^Pielou’s evenness.

^e^Significant differences between OD and MBR (*P* < 0.05).

**Table 2:** Significance tests of the differences of functional genes of the two systems based on non-parametric multivariate analysis of variance (ADONIS), analysis of similarity (ANOSIM) and multiple response permutation procedure (MRPP).

	ADONIS		ANOSIM		MRPP	
	R	*P*	*F*	**P**		*P*
Overall functional genes	0.171	**<0.001**	0.386	**<0.001**	0.13	**<0.001**
Carbon cycling genes	0.219	**<0.001**	0.483	**<0.001**	0.119	**<0.001**
Organic remediation	0.198	**<0.001**	0.422	**<0.001**	0.109	**<0.001**
Nitrogen cycling genes	0.22	**<0.001**	0.462	**<0.001**	0.125	**<0.001**
Phosphorus cycling genes	0.223	**<0.001**	0.489	**<0.001**	0.132	**<0.001**

**Table 3:** Major topological properties of the empirical association networks of core genes involved in the cycling of carbon, nitrogen and phosphorus in the MBR and OD systems and their associated random networks.

System	Empirical networks			r of scale free^c^ (significance)	Avg connectivity	Avg geodesic distance	Avg clustering coefficient	Modularity (No. of modules)	Transitivity (Trans)	Random networks^d^		
	No. of original genes^a^	S_t_	Network size (n)^b^							Avg geodesic distance ± SD	Avg clustering distance ± SD	Avg modularity ± SD
OD	3669	0.95	1426	0.91 (<0.001)	2.05	0.93^e^	0.13^e^	0.94^e^ (295)	0.349^e^	4.66 ± 0.24	0.001 ± 0.001	0.85 ± 0.00
MBR	3261	0.96	1116	0.95 (<0.001)	3.12	2.59^e^	0.19^e^	0.82^e^ (200)	0.255^e^	3.94 ± 0.13	0.006 ± 0.002	0.61 ± 0.00

^a^The number of genes originally used for network construction using the RMT-based approach.

^b^The number of nodes in a network.

^c^The correlation coefficient (*r*) of the linear relationship in log[*P(k)*] ~ -*γ*log(*k*), where *P(k)* is the fraction of connectivity *k* and *γ* is a constant.

^d^The random networks were generated by rewiring all of the links of a network with the identical numbers of nodes and links to the corresponding empirical network.

^e^Significant difference (*P* < 0.001) between the networks of OD and MBR.
