# Supplementary material for: Diversity and interactions of microbial functional genes under differing environmental conditions: insights from a membrane bioreactor and an oxidation ditch
Source: Sci Rep. 2016 Jan 8;6:18509. doi: 10.1038/srep18509 (PMC4705467; doi:10.1038/srep18509)
Supplement: Supplementary Information [file srep18509-s1.doc]

*Supporting information*

**Diversity and interactions of microbial functional genes under differing environmental conditions: insights from a membrane bioreactor and an oxidation ditch**

Yu Xia^1^, Man Hu^1^, Xianghua Wen^1*^, Xiaohui Wang^1^, Yunfeng Yang^1^, Jizhong Zhou^1, 2^

^1^Environmental Simulation and Pollution Control State Key Joint Laboratory, School of Environment, Tsinghua University, 100084, Beijing, P.R. China

^2^Institute for Environmental Genomics and Department of Botany and Microbiology, University of Oklahoma, Norman, OK, USA

*Corresponding author

E-mail: [xhwen@tsinghua.edu.cn](mailto:xhwen@tsinghua.edu.cn)

Tel: +86-10-62772837

Fax: +86-10-62771472

**Text S1:** Topological features of the two constructed association networks

The node degree distributions (connectivity) of the constructed association networks fit the power law model well, with typical correlation values of ~0.91 to ~0.95, respectively (Table 3), indicating that the networks in these microbial communities exhibited scale-free behavior, at least approximately. Also, the path length and clustering coefficients of the two constructed networks were significantly different from those of the corresponding random networks with the same network size and average number of links as revealed by the *Z*-test (Table 3), indicating that the correlation networks of core genes involved in the cycling of carbon, nitrogen and phosphorus showed typical small-world characteristics. In the networks examined here, a module is a group of functional genes that are highly connected among themselves but have few connections with the functional genes belonging to other modules. The two networks examined were modular, with a significantly higher modularity (M) than those from the corresponding random networks (Table 3).

**Tables**

**Table S1.** Comparison of treatment processes and environmental variables of the two systems and their possible impacts on the overall functional genes.

|  | **MBR** | **OD** | **Possible impacts on microbial community** |
| --- | --- | --- | --- |
| Treatment process | 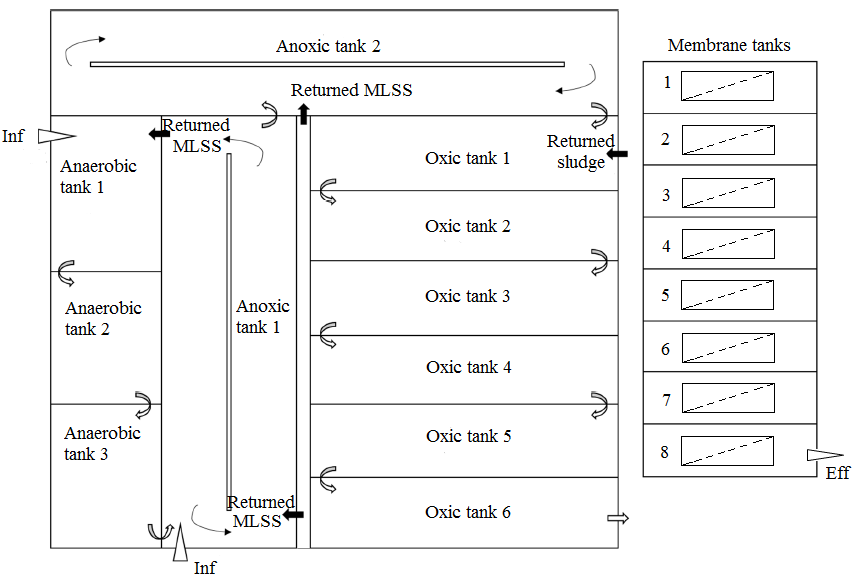 | 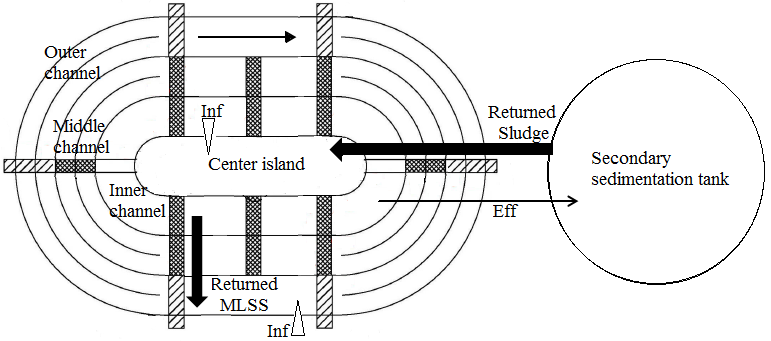 | Contributing to the co-existence of a large number of functional genes in several categories in the systems, for example, carbon cycling, nitrogen cycling and phosphorus cycling. |
|  | The A^2^O-MBR system contained anaerobic, anoxic and oxic tanks, which were designed mainly for the biological removal of carbon, nitrogen and phosphorus pollutants. The membrane tank was used for the liquid-solid separation and guaranteeing a high quality effluent. The system was generally operated at a high MLSS concentration and long SRT. | The orbal oxidation ditch had three concentric channels. The outer channel contained anoxic and aerobic zones, and even anaerobic condition could be attained in some part.  DO gradually decreases along the channel from the back of  the aeration disc [upstream (DO > 1.0mg/L)] to  the front of the next aeration disc [downstream (DO  ≈ 0)]. The middle channel also included anoxic and aerobic zones. The inner channel could be classified as an aeration reactor. An internal recycle was applied (from the inner channel to the outer channel) to promote nitrogen removal performance. The center island was reconstructed to an aerated regeneration tank of recycle sludge. |  |
|  |  |  |  |
| Influent characteristics | Municipal wastewater (I : D = 0.4 : 0.6) | Municipal wastewater (I : D = 0.4 : 0.6) | Promoting functional gene similarity of the systems |
|  |  |  |  |
| Sludge inoculum | The OD sludge | Activated sludge from a full-scale municipal wastewater treatment system | Promoting functional gene similarity of the systems |
|  |  |  |  |
| DO concentrations | 2.95 ± 0.46 mg/L (aeration tank) | 1.69 ± 0.51 mg/L (inner channel) | Higher aeration rate of the MBR may select against denitrifiers and *ppx* genes |
|  |  |  |  |
| MLSS concentrations | ~ 7,000 mg/L (aeration tank) | ~ 4,500 mg/L (inner channel) | Higher MLSS of the MBR resulting in lower F/M ratios may favor *K*-strategists like *Nitrosospira*, select against denitrifers and promote *phytase* genes in the MBR system. Also, it resulted in the higher polysaccharides of the supernatant, which may contribute to the higher abundance of carbon degradation genes of the MBR system. |
|  |  |  |  |
| SRT | ~ 20.5 d | ~ 16.2 d | Longer SRT may be a possible contributor of the higher abundance of carbon degradation genes in the MBR system |

**Table S2.** Wastewater characteristics of the two treatment systems during the sampling days.

| Date | COD (mg/l) ^a^ | NH_4_^+^-N (mg/l) ^a^ | TN (mg/l) ^a^ | TP (mg/l) ^a^ | Temperature (℃) | pH |
| --- | --- | --- | --- | --- | --- | --- |
| 2011/4/10 | 418.3 ± 1.0 | 33.4 ± 0.2 | 44.6 ± 1.0 | 4.5 ± 0.2 | 19.4 ± 0.1 | 7.32 ± 0.07 |
| 2011/4/11 | 464.7 ± 2.0 | 30.2 ± 0.3 | 43.3 ± 1.3 | 8.1 ± 0.1 | 19.9 ± 0.2 | 7.16 ± 0.13 |
| 2011/4/12 | 573.2 ± 2.5 | 40.4 ± 0.2 | 50.5 ± 0.8 | 4.8 ± 0.2 | 20.0 ± 1.0 | 7.07 ± 0.02 |
| 2011/4/13 | 431.2 ± 0.8 | 44.3 ± 0.0 | 56.0 ± 0.5 | 3.1 ± 0.2 | 20.1 ± 0.5 | 7.21 ± 0.15 |
| 2011/4/14 | 381.0 ± 1.0 | 37.5 ± 0.3 | 44.2 ± 1.2 | 4.8± 0.3 | 21.7 ± 1.0 | 7.23 ± 0.05 |
| 2011/4/15 | 337.3 ± 1.4 | 30.8 ± 0.1 | 40.3 ± 0.5 | 4.2 ± 0.1 | 18.9 ± 0 | 7.46 ± 0.12 |
| 2011/4/16 | 323.2 ± 1.0 | 28.8 ± 0.4 | 35.4 ± 0.5 | 4.6 ± 0.2 | 20.5 ± 0.3 | 7.19 ± 0.03 |
| 2011/4/17 | 456.5 ± 2.1 | 41.3 ± 0.2 | 48.5 ± 1.5 | 6.1 ± 0.3 | 20.2 ± 0.5 | 7.09 ± 0.07 |
| 2011/4/18 | 295.3 ± 0.2 | 32.4 ± 0.1 | 45.3 ± 0.7 | 5.2 ± 0.2 | 19.1 ± 0 | 7.10 ± 0.06 |
| 2011/4/19 | 380.5 ± 1.0 | 28.2 ± 0.2 | 40.5 ± 0.3 | 4.5 ± 0.1 | 19.1 ± 0 | 7.10 ± 0.13 |
| 2011/4/20 | 410.0 ± 1.3 | 32.3 ± 0.3 | 46.1 ± 0.2 | 6.3 ± 0.2 | 19.4 ± 0.3 | 7.45 ± 0.05 |
| 2011/4/21 | 322.1 ± 1.8 | 29.6 ± 0.2 | 35.3 ± 0.5 | 4.9 ± 0.2 | 18.5 ± 0 | 7.36 ± 0.04 |

a Concentrations of COD, NH_4_^+^-N, TN and TP of the influents of the two systems (24-h composite samples).

**Table S3.** Characteristics of the effluents and parameters of each bioreactor during the sampling days.

| Sample | COD eff ^a^  (mg/l) | NH_4_^+^-N eff ^a^  (mg/l) | TN eff ^a^ (mg/l) | TP eff ^a^ (mg/l) | | Temperature ^b^ (℃) | | DO ^b^(mg/l) | | | pH ^b^ | | Removal efficiency (%) | | | |
| --- | --- | --- | --- | --- | --- | --- | --- | --- | --- | --- | --- | --- | --- | --- | --- | --- |
|  |  |  |  |  |  |  |  |  |  |  |  |  | COD | TN | TP | NH_4_^+^-N |
| OD1 | 32.2 ± 0.3 | 0.382 ± 0.010 | 12.3 ± 0.5 | | 0.2 ± 0 | | 17.7 ± 0.5 | | 1.70 | 6.94 ± 0.04 | | 92.3 | | 72.4 | 97.8 | 98.9 |
| OD2 | 27.0 ± 0.2 | 0.424 ± 0.003 | 13.4 ± 0.2 | | 0.1 ± 0 | | 18.2 ± 1.0 | | 2.10 | 7.21 ± 0.10 | | 94.2 | | 69.1 | 98.8 | 98.6 |
| OD3 | 19.5 ± 0.5 | 0.416 ± 0 | 11.0 ± 0.2 | | 0.1 ± 0.1 | | 18.6 ± 0.3 | | 3.00 | 7.04 ± 0.12 | | 96.6 | | 78.2 | 95.8 | 99.0 |
| OD4 | 42.9 ± 1.5 | 0.454 ± 0.020 | 14.7 ± 0.4 | | 0.1 ± 0 | | 18.8 ± 0.2 | | 1.58 | 7.01 ± 0.05 | | 90.1 | | 73.8 | 96.8 | 99.0 |
| OD5 | 33.5 ± 0.3 | 0.53 ± 0.005 | 11.6 ± 0.2 | | 0.1 ± 0.1 | | 19.7 ± 0.2 | | 1.80 | 7.03 ± 0.06 | | 91.2 | | 73.8 | 97.9 | 98.6 |
| OD6 | 40.9 ± 1.5 | 0.452 ± 0.003 | 14.2 ± 0.1 | | 0.1 ± 0.1 | | 19.3 ± 0.3 | | 1.65 | 7.15 ± 0.15 | | 87.9 | | 64.8 | 97.6 | 98.5 |
| OD7 | 32.8 ± 0.8 | 0.28 ± 0 | 12.8 ± 0.2 | | 0.1 ± 0.2 | | 22.5 ± 0.5 | | 1.17 | 6.93 ± 0.02 | | 89.9 | | 63.8 | 97.8 | 99.0 |
| OD8 | 21.7 ± 0.2 | 0.4 ± 0.010 | 11.2 ± 0.3 | | 0.1 ± 0 | | 19.5 ± 1.2 | | 1.39 | 7.06 ± 0.04 | | 95.2 | | 76.9 | 98.4 | 99.0 |
| OD9 | 26.0 ± 0.2 | 0.354 ± 0.003 | 11.8 ± 0.2 | | 0.2 ± 0.1 | | 19.1 ± 0.3 | | 1.59 | 7.08 ± 0.03 | | 91.2 | | 74.0 | 98.1 | 98.9 |
| OD10 | 42.9 ± 0.4 | 0.448 ± 0.002 | 12.6 ± 0.1 | | 0.1 ± 0.2 | | 19.3 ± 0.2 | | 1.53 | 6.94 ± 0.03 | | 88.7 | | 68.9 | 97.8 | 98.4 |
| OD11 | 29.8 ± 0.3 | 1.05 ± 0 | 12.0 ± 0.3 | | 0.2 ± 0.1 | | 19.5 ± 0 | | 1.68 | 7.14 ± 0.09 | | 92.7 | | 74.0 | 96.8 | 96.7 |
| OD12 | 25.5 ± 0.4 | 0.751 ± 0.002 | 11.0 ± 0.3 | | 0.1 ± 0 | | 18.9 ± 0 | | 1.13 | 7.10 ± 0.05 | | 92.1 | | 68.8 | 95.9 | 97.5 |
| MBR1 | 29.3 ± 0.4 | 0.396 ± 0.003 | 14.1 ± 0.3 | | 0.1 ± 0.1 | | 17.8 ± 0.2 | | 1.67 | 6.99 ± 0.03 | | 93.0 | | 68.4 | 95.6 | 98.8 |
| MBR2 | 20.2 ± 0.5 | 0.606 ± 0.005 | 14.0 ± 0.1 | | 0.1 ± 0 | | 17.9 ± 0.3 | | 2.98 | 6.88 ± 0.04 | | 95.7 | | 67.7 | 98.8 | 98.0 |
| MBR3 | 13.6 ± 0.4 | 0.644 ± 0 | 13.0 ± 0.2 | | 0.2 ± 0 | | 18.1 ± 0.9 | | 3.10 | 6.90 ± 0.11 | | 97.6 | | 74.3 | 97.9 | 98.4 |
| MBR4 | 25.8 ± 0.3 | 0.475 ± 0.001 | 14.8 ± 0.2 | | 0.1 ± 0.1 | | 19.4 ± 1.4 | | 3.07 | 6.87 ± 0.06 | | 94.0 | | 73.6 | 96.8 | 98.9 |
| MBR5 | 29.3 ± 0.5 | 0.613 ± 0.001 | 17.7 ± 0.4 | | 0.1 ± 0 | | 19.7 ± 0.3 | | 3.30 | 6.85 ± 0.03 | | 92.3 | | 60.0 | 97.9 | 98.4 |
| MBR6 | 29.9 ± 0.8 | 0.559 ± 0.002 | 15.2 ± 0.7 | | 0.1 ± 0.1 | | 19.6 ± 0.3 | | 3.53 | 7.02 ± 0.03 | | 91.1 | | 62.3 | 97.6 | 98.2 |
| MBR7 | 31.8 ± 0.4 | 0.98 ± 0.004 | 15.5 ± 0.3 | | 0.1 ± 0 | | 19.3 ± 0.7 | | 3.14 | 6.78 ± 0.05 | | 90.2 | | 56.2 | 97.8 | 96.6 |
| MBR8 | 19.5 ± 0.2 | 0.374 ± 0.003 | 11.8 ± 0.2 | | 0.1 ± 0 | | 19.6 ± 0.2 | | 2.92 | 6.96 ± 0.05 | | 95.7 | | 75.7 | 98.4 | 99.1 |
| MBR9 | 26.0 ± 0.1 | 0.901 ± 0.002 | 13.1 ± 0.1 | | 0.1 ± 0 | | 19.7 ± 0.9 | | 3.18 | 6.97 ± 0.10 | | 91.2 | | 71.1 | 96.2 | 97.2 |
| MBR10 | 25.8 ± 0.3 | 0.455 ± 0.005 | 14.4 ± 0.2 | | 0.1 ± 0.1 | | 19.6 ± 0.3 | | 2.63 | 6.90 ± 0.03 | | 93.2 | | 64.4 | 97.8 | 98.4 |
| MBR11 | 21.3 ± 0.8 | 0.409 ± 0 | 11.6 ± 0.2 | | 0.2 ± 0.1 | | 19.4 ± 0.1 | | 2.83 | 6.99 ± 0.05 | | 94.8 | | 74.8 | 96.8 | 98.7 |
| MBR12 | 22.5 ± 1.0 | 0.473 ± 0.3 | 15.0 ± 0.3 | | 0.2 ± 0 | | 19.4 ± 0.1 | | 3.02 | 6.98 ± 0.12 | | 93.0 | | 57.5 | 98.0 | 98.4 |

a COD eff , NH_4_^+^-N eff, TN eff and TP eff: Concentrations of COD, NH_4_^+^-N, TN and TP of the effluents of the two systems (24-h composite samples).

b Temperature, DO and pH: data measured in the aeration tanks of the OD system and of the A^2^O-MBR system.

**Table S4.** The genes present in all the sampling days of each system.

| Gene Name | Category | Subcategory | Number_MBR_ | Number_OD_ |
| --- | --- | --- | --- | --- |
| SMR_antibiotics | Antibiotic resistance | transporter | 193 | 203 |
| MFS_antibiotic | Antibiotic resistance | transporter | 143 | 152 |
| B_lactamase_C | Antibiotic resistance | Beta-lactamases | 75 | 88 |
| Tet | Antibiotic resistance | other | 55 | 53 |
| B_lactamase_A | Antibiotic resistance | Beta-lactamases | 30 | 35 |
| MATE_antibiotic | Antibiotic resistance | transporter | 28 | 32 |
| ABC_antibiotic_transporter | Antibiotic resistance | transporter | 23 | 22 |
| Mex | Antibiotic resistance | transporter | 15 | 16 |
| B_lactamase | Antibiotic resistance | Beta-lactamases | 7 | 10 |
| Van | Antibiotic resistance | other | 1 | 2 |
| B_lactamase_B | Antibiotic resistance | Beta-lactamases | 0 | 1 |
| contractile_tail_sheath_protein | Bacteria phage | STRUCTURAL | 5 | 6 |
| major_capsid_protein | Bacteria phage | STRUCTURAL | 3 | 6 |
| DNA_polymerase_type_I | Bacteria phage | REPLICATION | 4 | 5 |
| endolysin_transglycosylase | Bacteria phage | LYSIS | 5 | 5 |
| integrase_tyrosine | Bacteria phage | REPLICATION | 5 | 5 |
| single_strand_annealing_protein | Bacteria phage | REPLICATION | 5 | 5 |
| helicase_family_4_DnaB_like | Bacteria phage | REPLICATION | 3 | 3 |
| lysin | Bacteria phage | LYSIS | 2 | 3 |
| non_contractile_major_tail_protein | Bacteria phage | STRUCTURAL | 2 | 3 |
| RNA_dependent__RNA_polymerase | Bacteria phage | REPLICATION | 1 | 3 |
| T4_recomb_endonuclease | Bacteria phage | REPLICATION | 1 | 3 |
| terminase_large_subunit | Bacteria phage | REPLICATION | 2 | 3 |
| holin_type_2 | Bacteria phage | LYSIS | 2 | 2 |
| Host_recognition_T2_type | Bacteria phage | HOST RECOGNITION/STRUCTURAL | 2 | 2 |
| primase | Bacteria phage | REPLICATION | 1 | 2 |
| scaffold | Bacteria phage | STRUCTURAL | 4 | 2 |
| tape_measure_protein | Bacteria phage | STRUCTURAL | 2 | 2 |
| clamp_loader_T4_ATPase | Bacteria phage | REPLICATION | 1 | 1 |
| contractile_central_tail_tube_protein | Bacteria phage | STRUCTURAL | 2 | 1 |
| DNA_ligase | Bacteria phage | REPLICATION | 1 | 1 |
| helicase_family_4 | Bacteria phage | REPLICATION | 1 | 1 |
| Host_recognition_T4_type | Bacteria phage | HOST RECOGNITION/STRUCTURAL | 1 | 1 |
| initiator_protein__theta_replication | Bacteria phage | REPLICATION | 1 | 1 |
| T5_genome_internalization_A1 | Bacteria phage | REPLICATION | 1 | 1 |
| terminal_protein_linear_DNA_phage | Bacteria phage | REPLICATION | 1 | 1 |
| mycobacterium_LysB_lipase | Bacteria phage | LYSIS | 0 | 2 |
| helicase_family_1_Dda_like | Bacteria phage | REPLICATION | 0 | 1 |
| helicase_P4alpha_type | Bacteria phage | REPLICATION | 0 | 1 |
| holin_type1 | Bacteria phage | LYSIS | 0 | 1 |
| ssb | Bacteria phage | REPLICATION | 1 | 0 |
| endolysin_glycosidase | Bacteria phage | LYSIS | 1 | 0 |
| glycosyl_transferase_EPS | Bioleaching | EPS | 5 | 8 |
| cytochrome_c | Bioleaching | Electron transport | 3 | 5 |
| NADH_quinone_oxidoreductase | Bioleaching | Electron transport | 5 | 5 |
| biotin_carboxylase | Bioleaching | Carbon fixation | 3 | 4 |
| cbb | Bioleaching | Carbon fixation | 2 | 4 |
| heat_shock_protein | Bioleaching | other category | 4 | 4 |
| NADH_ubiquinone_oxidoreductase | Bioleaching | Electron transport | 3 | 4 |
| antioxidant | Bioleaching | Metal Resistance | 4 | 3 |
| ATP_synthase_F1_or_F0 | Bioleaching | other category | 1 | 3 |
| drug_resistance_transporter | Bioleaching | Antibiotic Resistance | 3 | 3 |
| Fe_S_cluster_binding_protein | Bioleaching | Electron transport | 3 | 3 |
| ferredoxin_oxidoreductase | Bioleaching | Electron transport | 5 | 3 |
| 4Fe_4S_ferredoxin | Bioleaching | Electron transport | 1 | 2 |
| ABC_transporter_III | Bioleaching | other category | 3 | 2 |
| capsular_polysaccharide_biosynthesis_protein_EPS | Bioleaching | EPS | 2 | 2 |
| chromium_resistance | Bioleaching | Metal Resistance | 2 | 2 |
| NAD_dependent_epimerase_dehydratase_family_protein_EPS | Bioleaching | EPS | 1 | 2 |
| nitrogen_fixation_genes | Bioleaching | Nitrogen | 1 | 2 |
| ABC_transporter_II | Bioleaching | other category | 1 | 1 |
| acetyl_CoA_carboxylase | Bioleaching | Carbon fixation | 1 | 1 |
| acpA_III | Bioleaching | other category | 1 | 1 |
| Adaptations_to_atypical_conditionsIII | Bioleaching | other category | 1 | 1 |
| arsenic_resistance_gene | Bioleaching | Metal Resistance | 2 | 1 |
| cadmium_resistance_gene | Bioleaching | Metal Resistance | 1 | 1 |
| cyc4 | Bioleaching | Electron transport | 1 | 1 |
| cytochrome_bd | Bioleaching | Electron transport | 1 | 1 |
| ferredoxin_I | Bioleaching | Electron transport | 1 | 1 |
| ferredoxin_II | Bioleaching | Electron transport | 1 | 1 |
| ferrochelatase | Bioleaching | Iron oxidation | 1 | 1 |
| formate_hydrogenlyase_complex | Bioleaching | Carbon fixation | 1 | 1 |
| GDP_EPS | Bioleaching | EPS | 1 | 1 |
| gyrB_gene | Bioleaching | other category | 1 | 1 |
| NDEDFP_EPS | Bioleaching | EPS | 1 | 1 |
| nif | Bioleaching | Nitrogen | 1 | 1 |
| related_thiosulfate_gene | Bioleaching | Sulphur | 1 | 1 |
| UDS_EPS | Bioleaching | EPS | 1 | 1 |
| UHGNAS_EPS | Bioleaching | EPS | 1 | 1 |
| universal_stress_Family_protein_III | Bioleaching | other category | 1 | 1 |
| mercury_resistant_gene | Bioleaching | Metal Resistance | 0 | 2 |
| aa3_type_cytochrome_oxidase | Bioleaching | Electron transport | 0 | 1 |
| GAPDH | Bioleaching | Carbon fixation | 0 | 1 |
| LPS_heptosyltransferase_EPS | Bioleaching | EPS | 0 | 1 |
| UTP_glucose_1_phosphate_uridylyltransferase_EPS | Bioleaching | EPS | 0 | 1 |
| thioredoxin | Bioleaching | Sulphur | 1 | 0 |
| pcc | Carbon cycling | Carbon fixation | 214 | 238 |
| amyA | Carbon cycling | Carbon degradation | 186 | 202 |
| AceB | Carbon cycling | Carbon degradation | 147 | 165 |
| endochitinase | Carbon cycling | Carbon degradation | 112 | 130 |
| AceA | Carbon cycling | Carbon degradation | 106 | 116 |
| vanA | Carbon cycling | Carbon degradation | 88 | 100 |
| CODH | Carbon cycling | Carbon fixation | 80 | 91 |
| ara | Carbon cycling | Carbon degradation | 88 | 89 |
| phenol_oxidase | Carbon cycling | Carbon degradation | 83 | 86 |
| acetylglucosaminidase | Carbon cycling | Carbon degradation | 87 | 80 |
| rubisco | Carbon cycling | Carbon fixation | 69 | 77 |
| xylA | Carbon cycling | Carbon degradation | 57 | 63 |
| pulA | Carbon cycling | Carbon degradation | 41 | 47 |
| cda | Carbon cycling | Carbon degradation | 39 | 45 |
| cellobiase | Carbon cycling | Carbon degradation | 45 | 44 |
| endoglucanase | Carbon cycling | Carbon degradation | 40 | 37 |
| exoglucanase | Carbon cycling | Carbon degradation | 30 | 32 |
| xylanase | Carbon cycling | Carbon degradation | 21 | 29 |
| CDH | Carbon cycling | Carbon degradation | 27 | 28 |
| glucoamylase | Carbon cycling | Carbon degradation | 24 | 27 |
| mannanase | Carbon cycling | Carbon degradation | 27 | 27 |
| mcrA | Carbon cycling | Methane | 21 | 24 |
| nplT | Carbon cycling | Carbon degradation | 20 | 24 |
| ara_fungi | Carbon cycling | Carbon degradation | 23 | 23 |
| glx | Carbon cycling | Carbon degradation | 21 | 23 |
| pmoA | Carbon cycling | Methane | 16 | 19 |
| exochitinase | Carbon cycling | Carbon degradation | 12 | 17 |
| pectinase | Carbon cycling | Carbon degradation | 9 | 16 |
| lip | Carbon cycling | Carbon degradation | 15 | 15 |
| aclB | Carbon cycling | Carbon fixation | 13 | 14 |
| mnp | Carbon cycling | Carbon degradation | 18 | 14 |
| limEH | Carbon cycling | Carbon degradation | 13 | 13 |
| vdh | Carbon cycling | Carbon degradation | 13 | 13 |
| FTHFS | Carbon cycling | Acetogenesis | 13 | 12 |
| mmoX | Carbon cycling | Methane | 7 | 8 |
| amyX | Carbon cycling | Carbon degradation | 2 | 2 |
| LMO | Carbon cycling | Carbon degradation | 2 | 2 |
| apu | Carbon cycling | Carbon degradation | 2 | 1 |
| AssA | Carbon cycling | Carbon degradation | 1 | 1 |
| camDCAB | Carbon cycling | Carbon degradation | 1 | 1 |
| isopullulanase | Carbon cycling | Carbon degradation | 2 | 1 |
| cytochrome | Energy process | Energy process | 114 | 130 |
| hydrogenase | Energy process | Energy process | 28 | 35 |
| P450 | Energy process | Energy process | 8 | 10 |
| Ni_Fe_hydrogenase | Energy process | Energy process | 5 | 7 |
| chitin_synthase | Fungi function | major biomolecule | 53 | 58 |
| ABC_multidrug_fungi | Fungi function | metal resistance | 51 | 55 |
| exoglucanase_fungi | Fungi function | carbon degradation | 27 | 33 |
| cutinase_fungi | Fungi function | carbon degradation | 21 | 23 |
| pec_CDeg | Fungi function | carbon degradation | 22 | 22 |
| cellobiase_fungi | Fungi function | carbon degradation | 19 | 21 |
| protease_serine_fungi | Fungi function | carbon degradation | 25 | 21 |
| endochitinase_fungi | Fungi function | carbon degradation | 14 | 19 |
| phospholipase_C_fungi | Fungi function | carbon degradation | 16 | 18 |
| metalloprotease_fungi | Fungi function | carbon degradation | 18 | 17 |
| amyA_fungi | Fungi function | carbon degradation | 10 | 15 |
| nitrate_reductase_fungi | Fungi function | nitrogen | 15 | 15 |
| xylanase_fungi | Fungi function | carbon degradation | 13 | 14 |
| exopolygalacturonase_fungi | Fungi function | carbon degradation | 14 | 13 |
| phospholipase_D_fungi | Fungi function | carbon degradation | 15 | 13 |
| p450aro | Fungi function | organic remediation | 10 | 12 |
| ferroxidase_high_affinity | Fungi function | iron | 8 | 11 |
| phospholipase_A2_fungi | Fungi function | carbon degradation | 14 | 11 |
| phospholipase_B_fungi | Fungi function | virulence | 9 | 11 |
| endopolygalacturonase_fungi | Fungi function | carbon degradation | 7 | 10 |
| glucoamylase_fungi | Fungi function | carbon degradation | 7 | 10 |
| pme_CDeg | Fungi function | carbon degradation | 11 | 9 |
| rgh_fungi | Fungi function | carbon degradation | 11 | 9 |
| chitin_deacetylase_fungi | Fungi function | carbon degradation | 6 | 8 |
| iron_permease_high_affinity | Fungi function | iron | 8 | 8 |
| sulfate_transporter | Fungi function | Sulfur | 5 | 8 |
| glnA_fungi | Fungi function | nitrogen | 8 | 7 |
| alpha_galactosidase_fungi | Fungi function | carbon degradation | 4 | 6 |
| Glucose_oxidase_fungi | Fungi function | carbon degradation | 6 | 6 |
| lactase_fungi | Fungi function | carbon degradation | 6 | 6 |
| vacuolar_iron_transport | Fungi function | iron | 7 | 6 |
| catalase_KatG_fungi | Fungi function | virulence | 6 | 5 |
| NRPS | Fungi function | iron | 6 | 5 |
| phytase_fungi | Fungi function | phosphorous | 5 | 5 |
| protease_aspartate_fungi | Fungi function | carbon degradation | 5 | 5 |
| sidA | Fungi function | iron | 5 | 5 |
| AceA_fungi | Fungi function | carbon degradation | 3 | 4 |
| AceB_fungi | Fungi function | carbon degradation | 3 | 4 |
| RgaE_fungi | Fungi function | carbon degradation | 5 | 4 |
| superoxide_dismutase_fungi | Fungi function | virulence | 1 | 4 |
| aflatoxin_aflM | Fungi function | toxin | 3 | 3 |
| ATP_sulphurylase | Fungi function | Sulfur | 2 | 3 |
| dmaW_ergot | Fungi function | toxin | 4 | 3 |
| hmgB_fungi | Fungi function | carbon degradation | 5 | 3 |
| hmgC_fungi | Fungi function | carbon degradation | 4 | 3 |
| MFS_fungi | Fungi function | virulence | 2 | 3 |
| nirK_fungi | Fungi function | nitrogen | 2 | 3 |
| siderophore_transporter | Fungi function | iron | 2 | 3 |
| Sulfhydryl_oxidase | Fungi function | carbon degradation | 2 | 3 |
| arsA_fungi | Fungi function | metal resistance | 4 | 2 |
| calcineurin_A_fungi | Fungi function | virulence | 1 | 2 |
| Dyp | Fungi function | Organic remediation | 1 | 2 |
| mannanase_fungi | Fungi function | carbon degradation | 2 | 2 |
| nbaC_fungi | Fungi function | carbon degradation | 2 | 2 |
| PAPS_reductase | Fungi function | Sulfur | 2 | 2 |
| pel_CDeg | Fungi function | carbon degradation | 2 | 2 |
| ppx_fungi | Fungi function | phosphorous | 2 | 2 |
| protease_cysteine_fungi | Fungi function | carbon degradation | 2 | 2 |
| acetylglucosaminidase_fungi | Fungi function | carbon degradation | 1 | 1 |
| Al_fungi | Fungi function | metal resistance | 1 | 1 |
| Cl_peroxidase_fungi | Fungi function | Organic remediation | 1 | 1 |
| exoinulinase_fungi | Fungi function | carbon degradation | 1 | 1 |
| ferric_reductase_transporter | Fungi function | iron | 1 | 1 |
| lipase_fungi | Fungi function | carbon degradation | 2 | 1 |
| p450nor | Fungi function | nitrogen | 2 | 1 |
| patulin_6MSAS | Fungi function | toxin | 1 | 1 |
| invertase_fungi | Fungi function | carbon degradation | 0 | 2 |
| aromatic_peroxygenase | Fungi function | Organic remediation | 0 | 1 |
| cah_fungi | Fungi function | carbon degradation | 2 | 0 |
| conidial_laccase | Fungi function | virulence | 1 | 0 |
| aflatoxin_aflN | Fungi function | toxin | 1 | 0 |
| CopA | Metal Resistance | Copper | 320 | 357 |
| ChrA | Metal Resistance | Chromium | 230 | 257 |
| czcA | Metal Resistance | Cadmium,Cobalt,Zinc | 172 | 202 |
| mer | Metal Resistance | Mercury | 134 | 153 |
| CadA | Metal Resistance | Cadmium | 125 | 143 |
| czcD | Metal Resistance | Cadmium,Cobalt,Zinc | 114 | 125 |
| ArsC | Metal Resistance | Arsenic | 98 | 121 |
| silC | Metal Resistance | Silver | 95 | 114 |
| ZntA | Metal Resistance | Zinc | 109 | 109 |
| TerC | Metal Resistance | Tellurium | 93 | 99 |
| TerD | Metal Resistance | Tellurium | 63 | 73 |
| TehB | Metal Resistance | Tellurium | 44 | 49 |
| TerZ | Metal Resistance | Tellurium | 25 | 36 |
| aoxB | Metal Resistance | Arsenic | 19 | 24 |
| merP | Metal Resistance | Mercury | 10 | 20 |
| merB | Metal Resistance | Mercury | 13 | 15 |
| arsB | Metal Resistance | Arsenic | 13 | 14 |
| Al | Metal Resistance | Aluminum | 12 | 13 |
| SilA | Metal Resistance | Silver | 14 | 13 |
| czcC | Metal Resistance | Cadmium,Cobalt,Zinc | 9 | 12 |
| arsM | Metal Resistance | Arsenic | 10 | 11 |
| cadBD | Metal Resistance | Cadmium | 9 | 11 |
| pbrA | Metal Resistance | Lead | 13 | 11 |
| nreB | Metal Resistance | Nickel | 10 | 10 |
| ArsA | Metal Resistance | Arsenic | 5 | 8 |
| silP | Metal Resistance | Silver | 5 | 8 |
| ZitB | Metal Resistance | Zinc | 8 | 8 |
| CorC | Metal Resistance | Cobalt | 9 | 7 |
| CusA | Metal Resistance | Copper | 3 | 6 |
| merT | Metal Resistance | Mercury | 2 | 6 |
| metC | Metal Resistance | Mercury | 5 | 6 |
| CusF | Metal Resistance | Copper | 4 | 5 |
| cnrA | Metal Resistance | Cobalt,Nickel | 3 | 4 |
| SmtA | Metal Resistance | Miscellaneous | 4 | 4 |
| CueO | Metal Resistance | Copper | 3 | 3 |
| pbrT | Metal Resistance | Lead | 1 | 3 |
| cnrC | Metal Resistance | Cobalt,Nickel | 1 | 1 |
| rcnA | Metal Resistance | Cobalt,Nickel | 1 | 1 |
| CusC | Metal Resistance | Copper | 0 | 1 |
| merG | Metal Resistance | Mercury | 0 | 1 |
| narG | Nitrogen | Denitrification | 283 | 345 |
| nifH | Nitrogen | Nitrogen fixation | 171 | 193 |
| amoA | Nitrogen | Nitrification | 159 | 180 |
| ureC | Nitrogen | Ammonification | 154 | 179 |
| nirK | Nitrogen | Denitrification | 87 | 114 |
| nirS | Nitrogen | Denitrification | 102 | 113 |
| nosZ | Nitrogen | Denitrification | 61 | 69 |
| nrfA | Nitrogen | Dissimilatory N reduction | 46 | 50 |
| napA | Nitrogen | Dissimilatory N reduction | 39 | 42 |
| nasA | Nitrogen | Assimilatory N reduction | 41 | 41 |
| NiR | Nitrogen | Assimilatory N reduction | 23 | 31 |
| norB | Nitrogen | Denitrification | 23 | 28 |
| NirB | Nitrogen | Assimilatory N reduction | 12 | 16 |
| gdh | Nitrogen | Ammonification | 9 | 11 |
| nirA | Nitrogen | Assimilatory N reduction | 6 | 10 |
| hao | Nitrogen | Nitrification | 5 | 5 |
| hzo | Nitrogen | Anammox | 2 | 2 |
| pimF | Organic Remediation | Aromatics | 482 | 566 |
| Catechol | Organic Remediation | Aromatics | 153 | 167 |
| nagG | Organic Remediation | Aromatics | 141 | 159 |
| nmoA | Organic Remediation | Aromatics | 141 | 159 |
| pcaG | Organic Remediation | Aromatics | 121 | 150 |
| phn | Organic Remediation | Herbicides related compound | 103 | 133 |
| tfdA | Organic Remediation | Aromatics | 114 | 122 |
| PobA | Organic Remediation | Aromatics | 92 | 104 |
| mdlC | Organic Remediation | Aromatics | 85 | 100 |
| GCoADH | Organic Remediation | Aromatics | 83 | 92 |
| nhh | Organic Remediation | Aromatics | 85 | 87 |
| nitA | Organic Remediation | Aromatics | 74 | 85 |
| mdlA | Organic Remediation | Aromatics | 67 | 84 |
| Arylest | Organic Remediation | Aromatics | 67 | 83 |
| linB | Organic Remediation | Pesticides related compound | 70 | 83 |
| hmgB | Organic Remediation | Aromatics | 66 | 80 |
| alkB | Organic Remediation | Other Hydrocarbons | 65 | 79 |
| hmgC | Organic Remediation | Aromatics | 64 | 75 |
| alkK | Organic Remediation | Others | 76 | 72 |
| hmgA | Organic Remediation | Aromatics | 71 | 72 |
| atzA | Organic Remediation | Herbicides related compound | 61 | 71 |
| nahA | Organic Remediation | Aromatics | 59 | 68 |
| PhaB | Organic Remediation | Aromatics | 55 | 68 |
| exaA | Organic Remediation | Chlorinated solvents | 64 | 66 |
| pheA | Organic Remediation | Aromatics | 57 | 66 |
| bphA | Organic Remediation | Aromatics | 55 | 64 |
| catB | Organic Remediation | Aromatics | 59 | 64 |
| proO | Organic Remediation | Aromatics | 52 | 61 |
| chnB | Organic Remediation | Other Hydrocarbons | 54 | 58 |
| akbF | Organic Remediation | Aromatics | 53 | 57 |
| tftH | Organic Remediation | Aromatics | 44 | 51 |
| bclA | Organic Remediation | Aromatics | 44 | 50 |
| nagI | Organic Remediation | Aromatics | 43 | 47 |
| phtA | Organic Remediation | Aromatics | 38 | 43 |
| catechol_B | Organic Remediation | Aromatics | 34 | 42 |
| dmsA | Organic Remediation | Others | 35 | 39 |
| atzC | Organic Remediation | Herbicides related compound | 33 | 38 |
| mauAB | Organic Remediation | Herbicides related compound | 35 | 37 |
| atzB | Organic Remediation | Herbicides related compound | 31 | 35 |
| linC | Organic Remediation | Pesticides related compound | 27 | 34 |
| pcpE | Organic Remediation | Herbicides related compound | 24 | 34 |
| tutFDG | Organic Remediation | Aromatics | 31 | 33 |
| cbdA | Organic Remediation | Aromatics | 34 | 32 |
| trzN | Organic Remediation | Herbicides related compound | 32 | 31 |
| bphC | Organic Remediation | Aromatics | 23 | 26 |
| pnbA | Organic Remediation | Aromatics | 19 | 25 |
| BADH | Organic Remediation | Aromatics | 22 | 24 |
| bco | Organic Remediation | Aromatics | 22 | 23 |
| ohbAB | Organic Remediation | Aromatics | 24 | 22 |
| benAB | Organic Remediation | Aromatics | 17 | 21 |
| rd | Organic Remediation | Chlorinated solvents | 17 | 21 |
| xylF | Organic Remediation | Aromatics | 17 | 21 |
| dehH109 | Organic Remediation | Chlorinated solvents | 21 | 20 |
| pcpB | Organic Remediation | Herbicides related compound | 20 | 20 |
| HcaB | Organic Remediation | Aromatics | 13 | 18 |
| xylC | Organic Remediation | Aromatics | 18 | 18 |
| xylJ | Organic Remediation | Aromatics | 16 | 18 |
| alkH | Organic Remediation | Other Hydrocarbons | 11 | 17 |
| mhpA | Organic Remediation | Aromatics | 15 | 17 |
| nagK | Organic Remediation | Aromatics | 13 | 17 |
| nitro | Organic Remediation | Others | 14 | 17 |
| phdCI | Organic Remediation | Aromatics | 17 | 17 |
| AmiE | Organic Remediation | Aromatics | 10 | 15 |
| cumA | Organic Remediation | Aromatics | 13 | 15 |
| GcdB | Organic Remediation | Aromatics | 14 | 15 |
| arhA | Organic Remediation | Aromatics | 12 | 14 |
| carA | Organic Remediation | Aromatics | 15 | 14 |
| mdlD | Organic Remediation | Aromatics | 14 | 14 |
| scnABC | Organic Remediation | Others | 13 | 14 |
| Xamo | Organic Remediation | Other Hydrocarbons | 13 | 14 |
| nbaC | Organic Remediation | Aromatics | 12 | 13 |
| xylG | Organic Remediation | Aromatics | 13 | 13 |
| atzD | Organic Remediation | Herbicides related compound | 9 | 12 |
| bphD | Organic Remediation | Aromatics | 11 | 12 |
| benD | Organic Remediation | Aromatics | 9 | 10 |
| nagL | Organic Remediation | Aromatics | 7 | 10 |
| oxdB | Organic Remediation | Aromatics | 9 | 10 |
| tfdB | Organic Remediation | Aromatics | 9 | 10 |
| ALN | Organic Remediation | Others | 4 | 9 |
| adpB | Organic Remediation | Pesticides related compound | 9 | 8 |
| BpH | Organic Remediation | Aromatics | 6 | 8 |
| cmuA | Organic Remediation | Chlorinated solvents | 6 | 8 |
| pchCF | Organic Remediation | Aromatics | 4 | 8 |
| ChnA | Organic Remediation | Other Hydrocarbons | 6 | 7 |
| dehH | Organic Remediation | Chlorinated solvents | 7 | 7 |
| alkJ | Organic Remediation | Other Hydrocarbons | 6 | 6 |
| Apc | Organic Remediation | Aromatics | 4 | 6 |
| BMO | Organic Remediation | Other Hydrocarbons | 6 | 6 |
| cpnA | Organic Remediation | Other Hydrocarbons | 5 | 6 |
| hdnO | Organic Remediation | Aromatics | 6 | 6 |
| mdlB | Organic Remediation | Aromatics | 6 | 6 |
| ophC | Organic Remediation | Aromatics | 6 | 6 |
| trzE | Organic Remediation | Herbicides related compound | 4 | 6 |
| xlnD | Organic Remediation | Aromatics | 6 | 6 |
| mhpB | Organic Remediation | Aromatics | 3 | 5 |
| mhpC | Organic Remediation | Aromatics | 4 | 5 |
| nahF | Organic Remediation | Aromatics | 4 | 5 |
| phdI | Organic Remediation | Aromatics | 4 | 5 |
| todC | Organic Remediation | Aromatics | 4 | 5 |
| tomA | Organic Remediation | Aromatics | 5 | 5 |
| bbsG | Organic Remediation | Aromatics | 4 | 4 |
| nicdehydr | Organic Remediation | Aromatics | 5 | 4 |
| PceA | Organic Remediation | Others | 5 | 4 |
| phtB | Organic Remediation | Aromatics | 5 | 4 |
| xylL | Organic Remediation | Aromatics | 3 | 4 |
| bphB | Organic Remediation | Aromatics | 2 | 3 |
| ChnE | Organic Remediation | Other Hydrocarbons | 3 | 3 |
| CMCI | Organic Remediation | Aromatics | 3 | 3 |
| cmtAb | Organic Remediation |  | 3 | 3 |
| fcbA | Organic Remediation | Aromatics | 4 | 3 |
| pcpA | Organic Remediation | Aromatics | 3 | 3 |
| bbs | Organic Remediation | Aromatics | 2 | 2 |
| cumB | Organic Remediation | Aromatics | 2 | 2 |
| dbdC | Organic Remediation |  | 1 | 2 |
| ebdABC | Organic Remediation | Aromatics | 2 | 2 |
| nbz | Organic Remediation | Aromatics | 1 | 2 |
| nbzB | Organic Remediation | Aromatics | 1 | 2 |
| nbzC | Organic Remediation | Aromatics | 3 | 2 |
| phdJ | Organic Remediation | Aromatics | 3 | 2 |
| Quinoline | Organic Remediation | Aromatics | 1 | 2 |
| sdsA | Organic Remediation | Others | 2 | 2 |
| tdnB | Organic Remediation | Aromatics | 2 | 2 |
| tmoABE | Organic Remediation | Aromatics | 1 | 2 |
| tphA | Organic Remediation | Aromatics | 1 | 2 |
| trzA | Organic Remediation | Herbicides related compound | 1 | 2 |
| xylXY | Organic Remediation | Aromatics | 2 | 2 |
| cbaA | Organic Remediation | Aromatics | 1 | 1 |
| CDD | Organic Remediation | Aromatics | 1 | 1 |
| CDO | Organic Remediation | Aromatics | 1 | 1 |
| chnC | Organic Remediation | Other Hydrocarbons | 2 | 1 |
| cumC | Organic Remediation | Aromatics | 1 | 1 |
| cumD | Organic Remediation |  | 1 | 1 |
| cymC | Organic Remediation | Aromatics | 1 | 1 |
| dfbA | Organic Remediation |  | 1 | 1 |
| dxnA | Organic Remediation | Aromatics | 1 | 1 |
| fcbB | Organic Remediation | Aromatics | 1 | 1 |
| flnB | Organic Remediation | Aromatics | 1 | 1 |
| linD | Organic Remediation | Pesticides related compound | 1 | 1 |
| mhqA | Organic Remediation | Aromatics | 1 | 1 |
| MSAD | Organic Remediation | Others | 1 | 1 |
| msmABCD | Organic Remediation | Others | 1 | 1 |
| nahB | Organic Remediation | Aromatics | 1 | 1 |
| nidA | Organic Remediation | Aromatics | 1 | 1 |
| Onr | Organic Remediation |  | 1 | 1 |
| phdA | Organic Remediation | Aromatics | 2 | 1 |
| phdG | Organic Remediation | Aromatics | 3 | 1 |
| POBMO | Organic Remediation | Aromatics | 2 | 1 |
| qorL | Organic Remediation | Aromatics | 3 | 1 |
| THL | Organic Remediation | Aromatics | 1 | 1 |
| thmAB | Organic Remediation | Others | 1 | 1 |
| tphB | Organic Remediation | Aromatics | 1 | 1 |
| xylM | Organic Remediation | Aromatics | 1 | 1 |
| badK | Organic Remediation | Aromatics | 0 | 1 |
| dbdD | Organic Remediation | Aromatics | 0 | 1 |
| pchA | Organic Remediation | Aromatics | 0 | 1 |
| cpnB | Organic Remediation | Other Hydrocarbons | 2 | 0 |
| gyrB | other category | gyrB | 248 | 286 |
| BchY | other category | chlorophyllide reductase | 19 | 24 |
| ppx | Phosphorus | Phosphorus utilization | 111 | 138 |
| ppk | Phosphorus | Phosphorus utilization | 82 | 95 |
| phytase | Phosphorus | Phosphorus utilization | 10 | 11 |
| cat_bac | soil_benifit |  | 125 | 139 |
| sped_bac | soil_benifit |  | 89 | 104 |
| phzF | soil_benifit |  | 71 | 78 |
| pcbC | soil_benifit |  | 42 | 48 |
| acc | soil_benifit |  | 46 | 45 |
| per_fun | soil_benifit | Carbon degradation | 32 | 44 |
| sid_bac | soil_benifit |  | 36 | 39 |
| spe | soil_benifit |  | 30 | 37 |
| sod_CuZn | soil_benifit |  | 29 | 33 |
| tre_fun | soil_benifit |  | 26 | 31 |
| cat_fun | soil_benifit |  | 15 | 12 |
| per_bac | soil_benifit |  | 6 | 11 |
| sid_fun | soil_benifit |  | 14 | 11 |
| sid_arc | soil_benifit |  | 10 | 10 |
| sod_nickel | soil_benifit |  | 6 | 8 |
| bacA | soil_benifit |  | 5 | 6 |
| lipo | soil_benifit |  | 3 | 6 |
| per_arc | soil_benifit |  | 4 | 6 |
| lgrD | soil_benifit |  | 3 | 5 |
| pabA | soil_benifit |  | 6 | 5 |
| cat_arc | soil_benifit |  | 4 | 4 |
| nep | soil_benifit |  | 3 | 3 |
| sped_ara | soil_benifit |  | 3 | 3 |
| lmbA | soil_benifit |  | 2 | 2 |
| cks | soil_benifit |  | 1 | 1 |
| eth | soil_benifit |  | 1 | 1 |
| prnB | soil_benifit |  | 1 | 1 |
| spaR | soil_benifit |  | 1 | 1 |
| sped_fungi | soil_benifit |  | 1 | 1 |
| strR | soil_benifit |  | 2 | 1 |
| INF1_elicitin_Oomycetes | soil_borne_pathogen | oomycete | 16 | 19 |
| papC | soil_borne_pathogen | adherence | 12 | 15 |
| Cyanide_hydratase_Fungi | soil_borne_pathogen | fungi | 12 | 13 |
| igaA | soil_borne_pathogen | regulation | 12 | 12 |
| necrosis_Oomycetes | soil_borne_pathogen | oomycete | 8 | 11 |
| Pg_Oomycetes | soil_borne_pathogen | oomycete | 6 | 9 |
| serine_protease_inhibitor_Oomycetes | soil_borne_pathogen | oomycete | 8 | 8 |
| hrcU | soil_borne_pathogen | secretion system | 6 | 5 |
| mgtB | soil_borne_pathogen | magnesium uptake | 5 | 5 |
| scytalone_dehydratase_Fungi | soil_borne_pathogen | fungi | 5 | 5 |
| hrpG | soil_borne_pathogen | secretion system | 2 | 4 |
| pectate_lyase_Oomycetes | soil_borne_pathogen | oomycete | 5 | 4 |
| RdRp_Nepovirus | soil_borne_pathogen | replication | 3 | 4 |
| vir | soil_borne_pathogen | secretion system | 4 | 4 |
| acsC | soil_borne_pathogen | iron uptake | 3 | 3 |
| CBEL_Oomycetes | soil_borne_pathogen | oomycete | 3 | 3 |
| coat_Aureusvirus | soil_borne_pathogen | structural | 2 | 3 |
| glucanase_inhibitor_Oomycetes | soil_borne_pathogen | oomycete | 3 | 3 |
| hrpB2 | soil_borne_pathogen | secretion system | 3 | 3 |
| hrpP | soil_borne_pathogen | secretion system | 2 | 3 |
| impJ | soil_borne_pathogen | secretion system | 2 | 3 |
| pat1 | soil_borne_pathogen | enzyme | 3 | 3 |
| PcF_Oomycetes | soil_borne_pathogen | oomycete | 3 | 3 |
| pectin_lyase_Oomycetes | soil_borne_pathogen | oomycete | 2 | 3 |
| txtA | soil_borne_pathogen | toxin | 1 | 3 |
| acsD | soil_borne_pathogen | iron uptake | 1 | 2 |
| avrBs1 | soil_borne_pathogen | secretion system | 1 | 2 |
| avrBs3 | soil_borne_pathogen | secretion system | 2 | 2 |
| coat_Necrovirus | soil_borne_pathogen | structural | 3 | 2 |
| coat_Nepovirus | soil_borne_pathogen | structural | 2 | 2 |
| coat_Tombusvirus | soil_borne_pathogen | structural | 3 | 2 |
| replicase_Carmovirus | soil_borne_pathogen | replication | 4 | 2 |
| replicase_Tombusvirus | soil_borne_pathogen | replication | 1 | 2 |
| xcpZ | soil_borne_pathogen |  | 2 | 2 |
| yopT | soil_borne_pathogen | enzyme | 2 | 2 |
| yscX | soil_borne_pathogen | secretion system | 1 | 2 |
| AVR1_Oomycetes | soil_borne_pathogen | oomycete | 1 | 1 |
| avrBs2 | soil_borne_pathogen |  | 1 | 1 |
| coat_Dianthovirus | soil_borne_pathogen | structural | 1 | 1 |
| coat_Pecluvirus | soil_borne_pathogen | structural | 1 | 1 |
| coat_Tritimovirus | soil_borne_pathogen | structural | 1 | 1 |
| cutinase_Oomycetes | soil_borne_pathogen | oomycete | 1 | 1 |
| dspE | soil_borne_pathogen | secretion system | 1 | 1 |
| enniatin_synthase_Fungi | soil_borne_pathogen | fungi | 1 | 1 |
| fyuA | soil_borne_pathogen | iron uptake | 1 | 1 |
| hrpX | soil_borne_pathogen | secretion system | 1 | 1 |
| hrpY2 | soil_borne_pathogen | secretion system | 1 | 1 |
| mip | soil_borne_pathogen | intracellular survival | 1 | 1 |
| movement_Furovirus | soil_borne_pathogen | virulence | 1 | 1 |
| pchG | soil_borne_pathogen |  | 1 | 1 |
| pinF1 | soil_borne_pathogen | enzyme | 1 | 1 |
| ptlB | soil_borne_pathogen | secretion system | 1 | 1 |
| replicase_Tobravirus | soil_borne_pathogen | replication | 1 | 1 |
| xcpY | soil_borne_pathogen |  | 1 | 1 |
| pec | soil_benifit | carbon degradation | 0 | 1 |
| impH | soil_borne_pathogen | secretion system | 0 | 2 |
| tom | soil_borne_pathogen | enzyme | 0 | 2 |
| AVR1a_Oomycetes | soil_borne_pathogen | oomycete | 0 | 1 |
| avrA | soil_borne_pathogen | secretion system | 0 | 1 |
| CRN_Oomycetes | soil_borne_pathogen | oomycete | 0 | 1 |
| yopD | soil_borne_pathogen | secretion system | 2 | 0 |
| coat_Carmovirus | soil_borne_pathogen | structural | 2 | 0 |
| xopD | soil_borne_pathogen | secretion system | 1 | 0 |
| TGB1_Pomovirus | soil_borne_pathogen | virulence | 1 | 0 |
| replicase_Necrovirus | soil_borne_pathogen | replication | 1 | 0 |
| replicase_Furovirus | soil_borne_pathogen | replication | 1 | 0 |
| coat_unassigned_Secoviridae | soil_borne_pathogen | structural | 1 | 0 |
| ATR13_Oomycetes | soil_borne_pathogen | oomycete | 1 | 0 |
| fnr | Stress | Oxygen stress | 411 | 468 |
| sigma_24 | Stress | Sigma factors | 345 | 398 |
| pstB | Stress | Phosphate limitation | 293 | 340 |
| glnA | Stress | Nitrogen limitation | 237 | 262 |
| sigma_70 | Stress | Sigma factors | 216 | 242 |
| obgE | Stress | Radiation stress | 167 | 197 |
| katE | Stress | Oxygen stress | 131 | 160 |
| pstC | Stress | Phosphate limitation | 113 | 143 |
| hrcA | Stress | Heat shock | 104 | 119 |
| pstA | Stress | Phosphate limitation | 104 | 113 |
| oxyR | Stress | Oxygen stress | 85 | 104 |
| ahpC | Stress | Oxygen stress | 86 | 100 |
| phoB | Stress | Phosphate limitation | 86 | 100 |
| sigma_32 | Stress | Sigma factors | 86 | 97 |
| narI | Stress | Oxygen limitation | 81 | 81 |
| clpC | Stress | Protein stress | 71 | 77 |
| grpE | Stress | Heat shock | 67 | 69 |
| proV | Stress | Osmotic stress | 58 | 62 |
| ahpF | Stress | Oxygen stress | 36 | 43 |
| narH | Stress | Oxygen limitation | 37 | 43 |
| sigma_38 | Stress | Sigma factors | 36 | 42 |
| pstS | Stress | Phosphate limitation | 34 | 39 |
| dnaK | Stress | Heat shock | 27 | 33 |
| phoA | Stress | Phosphate limitation | 28 | 28 |
| groEL | Stress | Heat shock | 21 | 25 |
| narJ | Stress | Oxygen limitation | 21 | 23 |
| cydB | Stress | Oxygen limitation | 22 | 21 |
| katA | Stress | Oxygen stress | 15 | 18 |
| cydA | Stress | Oxygen limitation | 13 | 17 |
| ctsR | Stress | Protein stress | 7 | 9 |
| proX | Stress | Osmotic stress | 3 | 8 |
| arcA | Stress | Oxygen limitation | 7 | 6 |
| bglH | Stress | Glucose limitation | 7 | 6 |
| GroES | Stress | Heat shock | 6 | 6 |
| arcB | Stress | Oxygen limitation | 4 | 5 |
| bglP | Stress | Glucose limitation | 2 | 5 |
| cspA | Stress | Cold shock | 3 | 4 |
| glnR | Stress | Nitrogen limitation | 3 | 4 |
| cspB | Stress | Cold shock | 3 | 3 |
| proW | Stress | Osmotic stress | 1 | 3 |
| perR | Stress | Oxygen stress | 2 | 2 |
| opuE | Stress | Osmotic stress | 6 | 1 |
| desR | Stress | Cold shock | 1 | 0 |
| desK | Stress | Cold shock | 1 | 0 |
| dsrA | Sulphur | sulfite reductase | 145 | 154 |
| sox | Sulphur | Sulphur oxidation | 107 | 140 |
| dsrB | Sulphur | sulfite reductase | 118 | 134 |
| CysJ | Sulphur | sulfite reductase | 96 | 109 |
| sir | Sulphur | sulfite reductase | 41 | 43 |
| fccAB | Sulphur | sulfide oxidation | 33 | 31 |
| APS_AprA | Sulphur | adenylylsulfate reductase | 18 | 24 |
| AprA | Sulphur | adenylylsulfate reductase | 20 | 23 |
| APS_AprB | Sulphur | adenylylsulfate reductase | 14 | 13 |
| sqr | Sulphur | sulfide oxidation | 6 | 4 |
| CysI | Sulphur | sulfite reductase | 3 | 2 |
| iro | virulence | Iron oxidation | 198 | 209 |
| pilin | virulence | pilin | 126 | 135 |
| hly | virulence | hemolysin | 72 | 84 |
| type_III_secretion | virulence | secretion | 50 | 54 |
| cap | virulence | capsule | 45 | 50 |
| srt | virulence | surface protein | 19 | 18 |
| inv | virulence | invasion | 10 | 13 |
| pap | virulence | adhesin | 8 | 13 |
| iuc | virulence | aerobactin | 11 | 9 |
| vip | virulence | virulence protein | 7 | 6 |
| colonization_factor | virulence | colonization factor | 1 | 2 |
| toxin | virulence | toxin | 2 | 2 |
| tcf | virulence |  | 1 | 1 |

**Table S5.** Information of module hubs and connector in each network. The two networks showed different module hubs (with different Genbank ID), and there was no connector detected within the OD system.

| System | Topological roles | Genbank ID | Gene | Organism | Gene category | Subcategory | Zi | Pi |
| --- | --- | --- | --- | --- | --- | --- | --- | --- |
| MBR | Module hubs | 269955722 | *AceB* | *Xylanimonas cellulosilytica* DSM 15894 | Carbon cycling | Carbon degradation | 3.789 | 0 |
|  |  | 118171974 | *CDH* | *Mycobacterium smegmatis* str. MC2 155 | Carbon cycling | Carbon degradation | 3.048 | 0.142 |
|  |  | 197772882 | *glx* | *Streptomyces pristinaespiralis* ATCC 25486 | Carbon cycling | Carbon degradation | 2.612 | 0 |
|  |  | 106889690 | *ara* | *Roseiflexus* sp. RS-1 | Carbon cycling | Carbon degradation | 2.862 | 0 |
|  |  | 254449746 | *AceA* | *Octadecabacter antarcticus* 238 | Carbon cycling | Carbon degradation | 2.674 | 0 |
|  |  | 226870051 | *AceB* | *Actinosynnema mirum* DSM 43827 | Carbon cycling | Carbon degradation | 2.514 | 0 |
|  |  | 170142908 | *AceB* | *Burkholderia graminis* C4D1M | Carbon cycling | Carbon degradation | 2.992 | 0 |
|  |  | 77690377 | *CODH* | *Rhodopseudomonas palustris* BisB5 | Carbon cycling | Carbon fixation | 2.834 | 0.244 |
|  |  | 6689448 | *hao* | unidentified anaerobic bacterium | Nitrogen | Nitrification | 2.64 | 0 |
|  |  | 22252768 | *nirK* | uncultured bacterium | Nitrogen | Denitrification | 2.716 | 0 |
|  |  | 99082987 | *nifH* | uncultured bacterium | Nitrogen | Nitrogen fixation | 3.994 | 0 |
|  |  | 85723765 | *nrfA* | *Syntrophus aciditrophicus* SB | Nitrogen | Dissimilatory N reduction | 3.181 | 0 |
|  | Connector | **108743469** | *ureC* | *Streptomyces kanamyceticus* | Nitrogen | Ammonification | -0.365 | 0.625 |
| OD | Module hubs | **156936409** | *amyA* | *Enterobacter sakazakii* ATCC BAA-894 | Carbon cycling | Carbon degradation | 4.065 | 0 |
|  |  | **169264** | *lip* | *Phanerochaete chrysosporium* | Carbon cycling | Carbon degradation | 3.872 | 0 |
|  |  | **119897408** | *AceA* | *Azoarcus* sp. BH72 | Carbon cycling | Carbon degradation | 3.283 | 0 |
|  |  | **119768086** | *amyA* | *Shewanella amazonensis* SB2B | Carbon cycling | Carbon degradation | 2.995 | 0 |
|  |  | **126236465** | *CDH* | *Mycobacterium* sp. JLS | Carbon cycling | Carbon degradation | 2.686 | 0 |
|  |  | **71554239** | *vdh* | *Pseudomonas syringae* pv. phaseolicola 1448A | Carbon cycling | Carbon degradation | 2.523 | 0 |
|  |  | **145223139** | *pcc* | *Mycobacterium gilvum* PYR-GCK | Carbon cycling | Carbon fixation | 2.715 | 0 |
|  |  | **146740636** | *rubisco* | *Cyanobium* sp. PCC 7001 | Carbon cycling | Carbon fixation | 2.66 | 0 |
|  |  | **56696014** | *ppx* | *Silicibacter pomeroyi* DSS-3 | Phosphorus | Phosphorus utilization | 3.294 | 0 |
|  |  | **78776860** | *ppx* | *Thiomicrospira denitrificans* ATCC 33889 | Phosphorus | Phosphorus utilization | 2.995 | 0 |
|  |  | **192897548** | *ppk* | uncultured bacterium | Phosphorus | Phosphorus utilization | 2.908 | 0 |
|  |  | **38373201** | *nosZ* | uncultured bacterium | Nitrogen | Denitrification | 4.186 | 0 |
|  |  | **29125948** | *nosZ* | uncultured soil bacterium | Nitrogen | Denitrification | 4.005 | 0.142 |
|  |  | **76667345** | *nifH* | uncultured nitrogen-fixing bacterium | Nitrogen | Nitrogen fixation | 3.394 | 0 |
|  |  | **89512298** | *nifH* | uncultured nitrogen-fixing bacterium | Nitrogen | Nitrogen fixation | 2.709 | 0 |
|  |  | **170027493** | *napA* | uncultured bacterium | Nitrogen | Dissimilatory N reduction | 3.446 | 0 |

**Table S6.** Mantel test on connectivity vs. the gene significances of environmental variables.

| Environmental factor | OD | | | MBR | |  |
| --- | --- | --- | --- | --- | --- | --- |
|  | r_M_^a^ | *p* | r_M_^a^ | | *p* | |
| pH | -0.01826 | 0.861 | 0.07937 | | **0.001** | |
| Temperature | -0.04042 | 0.995 | 0.0195 | | 0.145 | |
| DO | -0.03741 | 0.996 | 0.00827 | | 0.332 | |
| COD inf | -0.03651 | 0.996 | 0.00042 | | 0.467 | |
| TN inf | -0.07148 | 1 | 0.00654 | | 0.367 | |
| NH_3_-N inf | -0.03512 | 0.998 | -0.06618 | | 1 | |
| TP inf | -0.05201 | 1 | -0.07628 | | 1 | |

^a^ *r_M_*, Mantel’s correlation coefficient.
